# Development of glial restricted human neural stem cells for oligodendrocyte differentiation in vitro and in vivo

**DOI:** 10.1038/s41598-019-45247-3

**Published:** 2019-06-21

**Authors:** Sangita Biswas, Seung Hyuk Chung, Peng Jiang, Samaneh Dehghan, Wenbin Deng

**Affiliations:** 1 Department of Biochemistry and Molecular Medicine, School of Medicine, The University of California at Davis, Sacramento California, 95817 USA; 2 Institute for Pediatric Regenerative Medicine, Shriners Hospitals for Children, Sacramento California, 95817 USA; 3 Department of Pharmaceutical Sciences, Sun Yat-Sen University, Shenzhen, China; 4 Department of Oral Biology, College of Dentistry, The University of Illinois at Chicago, Chicago, Illinois 60612 USA

**Keywords:** Myelin biology and repair, Neural stem cells, Neural stem cells

## Abstract

In this study, we have developed highly expandable neural stem cells (NSCs) from HESCs and iPSCs that artificially express the oligodendrocyte (OL) specific transcription factor gene Zfp488. This is enough to restrict them to an exclusive oligodendrocyte progenitor cell (OPC) fate during differentiation *in vitro* and *in vivo*. During CNS development, Zfp488 is induced during the early stages of OL generation, and then again during terminal differentiation of OLs. Interestingly, the human ortholog Znf488, crucial for OL development in human, has been recently identified to function as a dorsoventral pattering regulator in the ventral spinal cord for the generation of P1, P2/pMN, and P2 neural progenitor domains. Forced expression of Zfp488 gene in human NSCs led to the robust generation of OLs and suppression of neuronal and astrocyte fate *in vitro* and *in vivo*. Zfp488 expressing NSC derived oligodendrocytes are functional and can myelinate rat dorsal root ganglion neurons *in vitro*, and form myelin in Shiverer mice brain *in vivo*. After transplantation near a site of demyelination, Zfp488 expressing hNSCs migrated to the lesion and differentiated into premyelinating OLs. A certain fraction also homed in the subventricular zone (SVZ). Zfp488-ZsGreen1-hNSC derived OLs formed compact myelin in Shiverer mice brain seen under the electron microscope. Transplanted human neural stem cells (NSC) that have the potential to differentiate into functional oligodendrocytes in response to remyelinating signals can be a powerful therapeutic intervention for disorders where oligodendrocyte (OL) replacement is beneficial.

## Introduction

Adult neural stem cells (NSCs) are operationally defined as multipotent, self-renewing cells in the CNS that are characterized by their ability to differentiate into neurons, astrocytes, and oligodendrocytes via exogenous stimuli from their microenvironment^1–4^. They reside in the forebrain and spinal cord niches as a proliferating adult precursor cell population and are maintained for life^5,6^. NSCs have been identified as an endogenous source of neuroregenerative cells that are mobilized during normal adult neurogenesis as well as after acute brain and spinal cord injury^7–9^, stroke^10^ and after acute^11^ or chronic demyelination^12,13^. However, the modest spontaneous functional recovery seen after brain injury^10^ indicates that endogenous NSCs have limited capacity for proliferation and differentiation under pathological conditions^14^.

A rational choice for neuro-repair, therefore, is to either transplant exogenous NSCs into the CNS with capabilities to efficiently migrate to the injury site and differentiate, or to promote endogenous NSC mediated neuroregeneration. Transplanted NSCs may aid neuroregeneration by at least three major mechanisms. First, they can provide direct replacement of dead neurons or glial cells; second, undifferentiated NSCs secrete neurotrophic factors that directly or indirectly save the injured tissue from secondary damage; and third, they can promote remyelination by endogenous OLs.

As a proof of principle, fetal human neural stem cells (hfNSCs) were tested for their capacity for OL replacement and remyelination as therapeutic interventions in rodent and primate models of MS and in rodent stroke models. These pioneering studies have shown that hfNSCs not only survived and migrated in the inflammatory environment of the Experimental Autoimmune Encephalomyelitis (EAE) and ischemic brain^15^, they were also able to attenuate the clinical signs of EAE^5,16,17^ and ischemia-induced CNS pathology^18^. These studies, however, pointed out that grafted hfNSCs although optimally equipped for homing to the lesions were mostly neuronogenic, and showed nominal differentiation into oligodendrocytes at the site of the lesion^18,19^. Thus, their beneficial actions were not via OL replacement. Other studies in rodents have shown that undifferentiated NSCs grafted into either intact or injured adult spinal cord either remain undifferentiated or differentiate to astrocytes^20,21^. Notably, these studies indicate that efficient oligodendrocyte replacement may not be achievable with naïve fetal NSCs, rather more lineage-restricted NSCs may be tested for developing future OL replacement strategies.

Alternate sources of NSCs are the human embryonic stem cells (hESCs) or induced pluripotent stem cells (iPSCs). HESC and iPSC derived NSCs have a robust capacity for self-renewal and multipotency *in vitro*^3^, and they have been suggested as promising candidates for cell therapy after brain injury^19,22^. They can successfully engraft after transplantation into the rodent or primate CNS, and they exhibit an innate capacity to migrate toward areas of CNS lesions, including ischemic, chemical, or neoplastic injury^7–11^. They can proliferate robustly, and they are amenability to viral or nonviral-mediated gene correction *in vitro* coupled to lack of tumorigenicity upon grafting where they survive, migrate, and differentiate^23–25^. Most importantly, transplanted NSCs can home in the niches, self-renew and provide a long-lasting supply of regenerative cells *in vivo*^19,22,23^. Thus, NSC transplantation may be a better strategy than direct OPC transplantation.

In the mouse EAE model, transplanted hESC-derived NSCs were shown to survive and migrate^26^, but exhibited limited oligodendrocyte differentiation, myelination or functional recovery^6^. Other studies found that the post-transplant beneficial effects in EAE were largely due to the immediate release of immune-modulatory agents at the site of the inflammatory lesions^27,28^ along with secretion of neuroprotective factors^17–20^ and promotion of endogenous OPC differentiation^29^. Transplantation of more fate-restricted NSCs, however, resulted in a better functional recovery in mouse EAE^30^ and SCI model compared to naïve NSCs^31^. Specifically, hESC derived NSCs differentiated to A2B5/NG2/PDGFR-α expressing OPCs have been transplanted in rat cervical SCI model and was found to be safe. This has initiated a phase I clinical trial for patients with thoracic SCI. Thus, it is of great interest to develop lineage-restricted NSCs as a source of progenitor cells capable of surviving and differentiating into functional OLs after transplantation into MS brain, and other conditions leading to OL death.

Thus, to generate and test human NSCs that are mainly committed to an oligodendroglial fate, we have developed hESCs and iPSCs derived hNSCs that constitutively express the transcription factor Zfp488. Zfp488 is a key regulator for the development of oligodendrocyte lineage cells and differentiation of OPCs^32,33^. Originally it was thought to be exclusively expressed in differentiating OL^32,33^. However, results from an RNA-sequencing transcriptome and data base of cells in the cerebral cortex show that Zfp488 gene is expressed in the newly formed oligodendrocytes and plays a crucial role in their terminal maturation^34^. A previous study from our laboratory showed that when adult mouse subventricular zone (SVZ) NSCs were transduced *in vivo* by injecting Zfp488 gene carrying retroviral particles (with ZsGreen1 reporter), they differentiated primarily into OPCs following cuprizone-induced demyelination^32^. Misexpression of Zfp488 in SVZ NSPCs significantly increased the number of mature ZsGreen1 positive OLs in the corpus callosum which translated to functional recovery of motor function deficit these mice^32^. This indicated that Zfp488 expression selectively directed the fate of mouse endogenous NSCs towards generating functional OL *in vivo*.

In the current study, we have tested the differentiation potential of hESCs and iPSC derived Zfp488-ZsGreen1-hNSCs compared to control hNSCs *in vitro* as well as *in vivo* in a mouse model of demyelination. We demonstrate that forced expression of Zfp488 in human NSCs directs them towards an OPC fate while largely suppressing astrocytes or neuronal fate. This OPCs can efficiently mature into OLs *in vivo* after transplantation in mouse LPC induced corpus callosum demyelination model. We also demonstrate that Zfp488-ZsGreen1-hNSCs can myelinate the shiverer mouse brain after transplantation and myelinate rat dorsal root ganglion (DRG) neurons *in vitro*. In contrast, control hNSCs largely remain undifferentiated *in vivo* (with some astrocyte differentiation) within the same time frame.

## Methods

### Generation of NSCs from hESCs and iPSC

**Figure 1:**
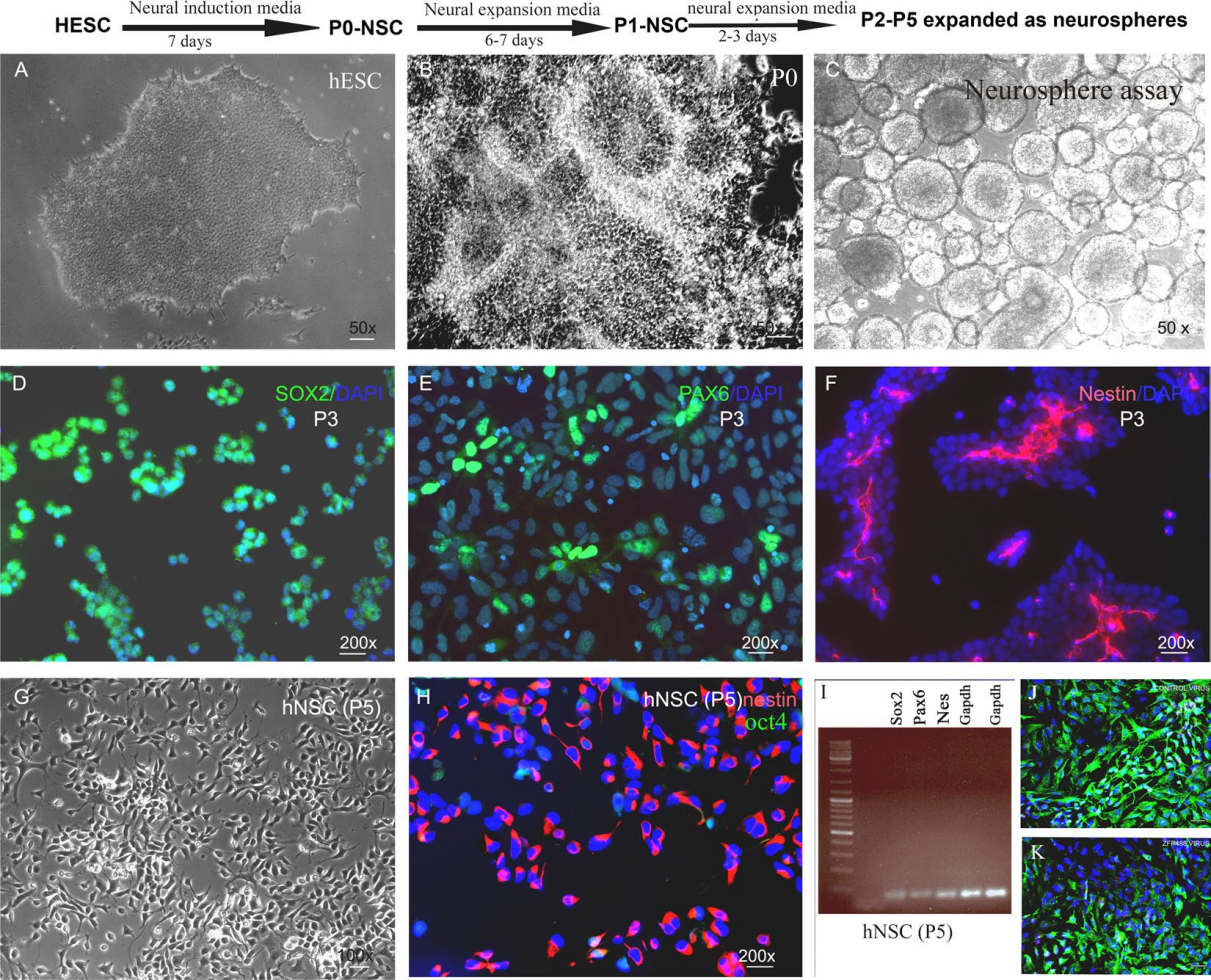
Adherent hESC–H9 colonies (**A**) treated with neural induction media for 7 days generated neural tube-like structures (**B**). After 7 days, cells were cultured as neurospheres in ultralow attachment plates (**C**). (**D**–**H**) Immunohistochemical analysis of hESC derived hNSCs. Passage 3 putative NSCs expressed neural stem cell markers SOX2, Pax6 and nestin (**D**,**E**,**H** respectively). Passage 5 (P5) hNSC represented cells with homogenous NSC morphology, and more than 97% of cells expressed nestin. These results were also confirmed by RT-PCR (**I**). Retrovirus transduction was confirmed by expression of ZsGreen1 fluorescence following differentiation of NSCs (**J**,**K** respectively).

hESC lines (H9 and BG01) and iPSCs (foreskin, clone 3)^35–37^ were a kind gift from Dr. James Thomson (University of Wisconsin-Madison, Madison, WI). IPSCs were generated in Thomson lab from human newborn foreskin fibroblasts (ATCC, USA) transduced with Oct4, SOX2, NANOG, and LIN28 containing lentiviruses. This resulted in constitutive expression of the transgenes. Cells were grown under feeder-free and serum-free conditions on hESC qualified Matrigel 4 mg/ml (Becton Dickinson, USA) coated plates in mTeSR1 media (Stemcell Technologies, USA). hNSCs were derived according to the protocol provided by the manufacturers (Thermo Fisher Scientific, USA). Briefly, on day 0 of splitting (15–20% confluency) (Fig. 1A), PSC Neural Induction Medium (Thermo Fisher Scientific, USA) was added into each well of 6-well plate of cells. Media was completely replaced on day 2, 4 and 6. On day 7 of neural induction cells are 90% confluent (Fig. 1B). Cells were passaged every 48–72 hours and expanded with neural expansion media (Thermo Fisher Scientific, USA). At passage 4–5, a population of NSCs with homogeneous morphology was obtained (Fig. 1G). These cells were either stocked or used for differentiation (population doubling time 48 hours).

### Characterization of hNSCs

Immunocytochemical staining and RT-PCR was used to determine the phenotypes of the NSCs. hNSCs plated on Matrigel-coated chamber slides were fixed with 4% paraformaldehyde/PBS and immunostained with NSC markers nestin, SOX2, PAX6, and pluripotency marker Oct4, using standard immunocytochemistry techniques. After washing with PBS, cells were permeabilized for 10 minutes in 0.1% Triton X-100 and incubated for 1 hour in blocking buffer (5% goat serum and 1% bovine serum albumin in PBS). Cells were then incubated overnight at 4 °C in primary antibodies diluted in blocking buffer (see Table 1 in supplementary document 1 for antibody dilutions and sources). Appropriate fluorescence-conjugated secondary antibodies (Alexa Fluor, Invitrogen, USA, dilution 1:1000) were used for single or double immunostaining.

### Generation of Zfp488-ZsGreen1-hNSCs and control ZsGreen1-hNSCs

Zfp488 and control lentiviral particles (both containing the ZsGreen1 reporter gene) used in this study were previously generated and tested for gene expression in our laboratory^32^. The mouse Zfp488 cDNA was a kind gift from Dr. Richard Lu (University of Texas Southwestern Medical Center, Dallas, TX, USA). The Zfp488 open reading frame was cloned into the pRetroX-IRES-ZsGreen1 vector (Clontech Laboratories, Mountain View, CA).The empty pRetroX-IRES vector expressing ZsGreen1 was used as a control. Retroviral particles were produced from pRetroX-Zfp488-IRES-ZsGreen1 and pRetroX-IRES-ZsGreen1 (control) at the Gene Transfer Vector Core, University of Iowa (Iowa City, IA). Titers for the concentrated stocks were obtained for both the viruses and were 2 × 10^8^ transducing units/ml. The Zfp488 gene is conserved between human and mouse (NCBI BLAST search). There is 56% homology in the amino acid sequence between the mouse and human Zfp488 protein and the zinc finger domains are conserved.

Passage 5 (P5) NSCs were infected with either Zfp488 or control retroviral vectors. NSCs were plated on Matrigel (4 mg/ml, Corning, USA) coated 6 well plates (0.3 × 10^6^ cells in each well). Zfp488 or control lentiviral particles (50 μl virus in 1 ml neural expansion media per well) and polybrene 6 μg/mL were added to each well. Plates were then sealed with parafilm and spun at 500 g for 30 min at 32 °C for increasing the efficiency of infection, followed by incubation in a CO_2_ incubator for 12–16 hours. Next day, the virus-containing media was discarded, and fresh neural expansion media was added. Cells were then allowed to recover for 4 hours, after which the second round of infection was performed for 6 hours using the same protocol. After infection cells were washed twice with PBS and cultured in neural expansion media until they were 90% confluent. Cells were split 1:3 in Matrigel-coated 6 well plates in neural expansion media for expansion. NSCs (P5-6) stocked at this stage in liquid nitrogen were successfully recovered for future use.

### Differentiation of control and Zfp488-ZsGreen1-hNSCs

Zfp488-ZsGreen1-hNSCs (P5-6) were evaluated for their potentials to differentiate into neurons or glial progenitor cells *in vitro*. Two different protocols, spontaneous as well as directed differentiation were used. Uninfected hNSCs, as well as hNSCs expressing only ZsGreen1 reporter (control hNSCs), were used as positive controls which could differentiate into neurons and astrocytes. For spontaneous differentiation, dissociated hNSCs were plated onto laminin (10 μg/ml, Sigma-Aldrich, USA) coated chamber slides at a density of 6.0 × 10^4^ cells per cm^2^. They were cultured under differentiation conditions (neurobasal media, 1x B27 supplement, and glutamax 2 mM) for 2–3 weeks without any growth factors.

For astroglial differentiation, dissociated NSCs were plated onto poly L-ornithine and laminin double-coated chamber slides at a density of 5.0 × 10^4^ cells per cm^2^ in an astrocyte differentiation medium (DMEM supplemented with 1% N2 supplement and 5% fetal bovine serum, Invitrogen USA) for 3 weeks to 7 weeks. Confluent cultures were passaged, and the medium was refreshed every 3–4 days. Differentiated cells were characterized by S100 beta at 3 weeks and GFAP immunostaining at 6 weeks (see supplementary document 1, figure B for the precise timeline of differentiation).

For directed OPC generation, dissociated Zfp488-ZsGreen1-hNSCs (P5-7) were plated onto poly-L-ornithine and laminin-coated coverslips at a density of 5.0 × 10^4^ cells per cm2 in OPC media 1 (DMEM/F12 medium, N2 supplement, 10 ng/ml basic fibroblast growth factor-2 (bFGF-2), 20 ng/ml epidermal growth factor (EGF) and 1 µM retinoic acid). After 1 week the cells were grown in OPC media 2 (DMEM/F12, 1% N2 supplement, 2% B27 supplement, 10 ng/ml PDGF-AA, insulin-like growth factor 10 ng/ml, NT3 10 ng/ml, and the sonic hedgehog agonist pumorphamine 1.0 µM, T3 30 ng/ml, biotin 100 ng/ml, and cAMP 1μM) for an additional 6–7 weeks. The early glial restricted precursor was assessed by A2B5 in some cases, and then by OPC markers PDGFR-α, NG2, Sox10, and Olig2 which were expressed between 3–4 weeks and used to characterize and quantify the cells. Half of the media was changed every other day throughout the protocol (see figure B in supplementary documents 1 for the timeline of OPC differentiation and characterization).

### Differentiation of FACS purified ZsGreen1/Zfp488^+^ OPCs

FAC sorted ZsGreen1 positive OPCs grown in suspension cultures were plated on Poly-L-ornithine and laminin-coated coverslips in OPC media 2 (DMEM/F12, N2 supplement100x, B27 supplement 50x, PDGF-AA 10 ng/mL, insulin-like growth factor 10 ng/ml, NT3 10 ng/ml, the sonic hedgehog (SHH) agonist pumorphamine 1.0 µM, T3 30 ng/ml, biotin 100 ng/ml, and cAMP 1μM). Media was changed every other day. After 5 weeks, the immunofluorescence study was performed on these cells to find the percentage of late OPCs by expression of O4 marker. Next, cells were differentiated in OL media (DMEM/F12, N2 supplement 100x, B27 supplement 50x, T3 30 ng/ml and ascorbic acid 20 mg/mL) only for one week. Then, immunofluorescence assay was performed on these cells to find the percentage of premyelinating oligodendrocytes by the expression of O1 and MBP (see figure B in supplementary documents for the timeline for differentiation).

### *In vivo* differentiation of Zfp488-ZsGreen1-hNSCs and control hNSCs after transplantation in a model of focal demyelination in mice

We compared the abilities of Zfp488-ZsGreen1-hNSCs and control hNSCs to engraft and differentiate *in vivo* following transplantation in a focal model of demyelination induced by a single injection of lysolecithin (LPC) in the corpus callosum of adult Rag-2 knockout mice (B6(Cg)-Rag2tm1.1Cgn/J, Jackson Laboratories, USA)^38^. Details of mice surgery are in supplemental document 1. In this model, demyelination occurs in about 48 hours and reaches maximum levels in 3–5 days^12^.

ZsGreen1 fluorescence and human nestin antibody were used to track the grafted cells. Double labeling immunofluorescence staining using antibodies against lineage-specific markers were used to compare the fate of Zfp488-ZsGreen1-hNSC and control ZsGreen1-NSCs (details of the antibodies are provided as Table 1 in supplementary documents). All animal procedures were approved by the IACUC committee of the University of California Davis. All experiments (*in vitro* and invivo including supplementary information) were performed in accordance with relevant guidelines and regulations.

### Myelination of shiverer mice brain by Zfp488-ZsGreen1-hNSCs

Shiverer mice (C3Fe.SWV-Mbpshi/J, The Jackson Laboratory, USA) lack myelin basic protein and myelin in the CNS^39,40^. Shiverer mice 8–10 weeks old (N = 3, each group) were anesthetized and injected with 50,000 iPSC derived Zfp488-ZsGreen1/PDGFR-α^+^ OPCs or sham injected with the same volume of phosphate buffered saline into the corpus callosum (surgical procedure and stereotaxic coordinates were identical with the LPC injections coordinates). Mice were administered cyclosporine 10 mg/kg, intraperitoneally daily for 8 weeks for immune suppression after which they were sacrificed. Brains were processed for electron microscope imaging. One-micron meter sections of the injected areas were analyzed for the formation of the multilayered compact myelin sheath. We could not derive enough control OPCs (with the empty virus) for transplantation using this protocol. All animal procedures were approved by the IACUC committee of UC Davis. All experiments (*in vitro* and invivo including supplementary information) were performed in accordance with relevant guidelines and regulations.

### Myelination of rat dorsal root ganglion (DRG) neurons by H9 derived Zfp488-ZsGreen1-hNSCs

Rat DRG (Lonza Inc, USA) were plated on collagen type I-coated coverslips (2–3 ganglion per coverslip) and allowed to mature for two weeks in primary neuronal growth media (Lonza Inc, USA). Non-neuronal cells (rat OPCs) were eliminated by cycling them three times with medium containing 5-fluorodeoxyuridine 0.1 μM and uridine 0.1 μM. Zfp488-ZsGreen1-iPSCs derived NSCs were differentiated to O4 expressing late OPCs. O4^+^ cells were identified by live immunostaining. O4 positive OPCs were then plated on top of the DRG neurons and cultured for three weeks in OL myelination medium (see supplementary document 1). After three to four weeks cells were fixed and immunostained for SMI312 (for neurofilament), myelin basic protein (MBP) or proteolipid peptide (PLP) for myelin and imaged by a confocal microscope (Nikon C1, USA). All experiments (including supplementary information) were performed in accordance with relevant guidelines and regulations set by UC Davis.

### Sox9 RT-PCR

Semiquantitative assay of Sox9 mRNA levels were done on Zfp488-ZsGreen1-hNSCs and control ZsGreen1-hNSCs immediately before differentiation, and after 3 days of differentiation in OPC media using the TaqMan gene expression system (N = 3 for each group). Presence of Sox9 protein was confirmed by immunohistochemistry using anti sox9 antibody (catalog # AF3075, R&D Systems, USA, N = 3, for each group).

### Statistical analysis

The differences in the numbers of Zfp488-ZsGreen1-hNSCs and control ZsGreen1-hNSCs that double labeled for OPC and astrocyte markers were compared by Students t-test. RT-PCR data were analyzed by one-way ANOVA followed by Students t-test.

## Results

### hNSCs derived from hESCs without embryoid body formation expressed neural stem cell markers

H9 hESC colonies (Fig. 1A) when exposed to neural induction media for 7 days changed morphology with the appearance of neuro rosette-like structures (Fig. 1B). After 1 week of neural induction, the putative P0-P1 NSCs expanded proficiently as monolayers in neural expansion media. hNSCs at P2-P5 were expanded as neurospheres in suspension culture (Fig. 1C). Neurospheres were then dissociated and expanded as adherent monolayer cultures. Putative hNSCs between passage 3–5 were immunostained for expression of NSC markers Sox2, nestin, Pax6 (Fig. 1D–F) and pluripotency marker Oct4. At P3, cells (<95%) were positive for Sox2 (Fig. 1D), PAX6 (<60%) (Fig. 1E), and nestin (~50%) (Fig. 1F). At P5 the plated cells showed homogeneous NSC morphology (Fig. 1G), and almost all cells expressed nestin (<97%) (Fig. 1H). Oct4^+^ cells were rarely seen (2.5 ± 0.6%). We confirmed the expression of sox2, pax6 and nestin in P5 hNSCs at the mRNA level by RT-PCR (See supplementary document Fig. A for iPSC and BG01 NSC data). The amplicon product sizes were as follows; nestin 81 bp, sox2 91 bp, pax6 86 bp, and GAPDH 157 bp (iPSC and BG01 derived P5-NSC data is included in supplementary document Table 2). Data for the characterization of iPSC derived NSCs are shown in supplementary document 1. Under our defined growth conditions, the hNSCs showed stable growth. The cells were highly proliferative in the presence of EGF and bFGF, and we speculate that billions of hNSCs could be produced over a period of 3–4 months. We expanded the isolated hNSCs cells for over 15 passages. Normally NSC generation by this protocol generates less than 5% OPCs *in vitro*. Their major fate is to differentiate into neuron (70–90%), and astrocytes (20–35%).

### Generation of Zfp488-ZsGreen1-hNSCs and control-hNSCs

A well-characterized repressor element present in stem cells prevents the expression of the Moloney Leukemia Retroviral -Long Terminal Repeat (MoMuLV-LTR) promoter prior to their differentiation into somatic or progenitor cells^41^. This unique property allowed us to “switch on” Zfp488 expression as the neural stem cells start to differentiate, but not during their self-renewal and expansion. Accordingly, after infecting the hNSCs, we confirmed that there was no expression of ZsGreen1 prior to the beginning of differentiation. Aliquots from the infected hNSCs were then differentiated by culturing them in DMEM/F12 with N2 and B27 supplements (without bFGF or EGF) to assess the efficiency of infection by counting the number of ZsGreen1 positive cells. These viruses were from the same batch that was previously generated and characterized in our laboratory^32^. Expression of Zfp488 was earlier confirmed and reported after a trial retroviral infection in 293T cells using both ZsGreen1 reporter fluorescence and western blots^32^. Transfection efficiency was assessed by differentiating aliquots of H9-derived control ZsGreen1-hNSCs or Zfp488-ZsGreen1-hNSCs spontaneously (by withdrawing growth factors EGF and bFGF from the media) to allow for ZsGreen1 expression. The number of ZsGreen1^+^ cells were found to be ~85% for control groups (Fig. 1J), and ~7% for the Zfp488 group. However, when Zfp488 infected cells were differentiated in OPC media for 3 weeks 70–80% cells became ZsGreen1 positive (Fig. 1K). Aliquots from these batches were frozen and recovered later for OPC differentiation and FAC sorting (via ZsGreen1 fluorescence) for *in vivo* transplantation studies in LPC-demyelination model in mouse. Zfp488-hNSCs were successfully derived from hiPSCs and BG01 hESC cells (Supplementary Figs 4 and 5 respectively).

### ZFP488 misexpression suppressed astrocytic fate *in vitro*

**Figure 2:**
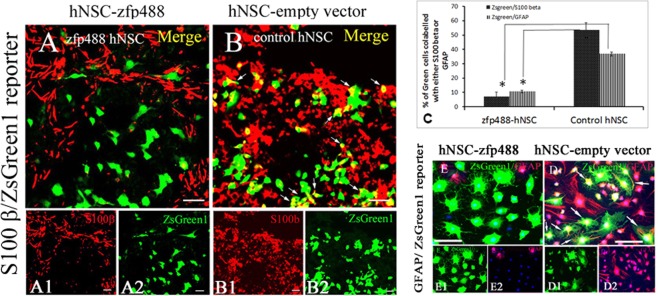
Astrocyte differentiation of control and Zfp488-ZsGreen1-hNSCs. (**A**) In Zfp488 group a low fraction of ZsGreen1^+^ cells expressed S100-beta (6.8 ± 3.5%) after 3 weeks of culture in astrocyte differentiation medium. (**B**) In contrast, half of (53.5 ± 7.2%) of ZsGreen1^+^ control hNSCs expressed S100-beta (see arrowheads). (**C**) Confocal image quantification of cells that co-labeled for ZsGreen1 and S100beta in the two groups showed that significantly higher number of the control hNSCs differentiated into S100-beta^+^ cells (Students unpaired t-test, P < 0.001). After 6 weeks of differentiation, GFAP^+^ astrocytes were detected in both groups (**D**,**E**). (**E**) Zfp488-ZsGreen1-hNSC had nominal GFAP expressing cells (10.6 ± 1.4%), whereas a significantly larger fraction of control ZsGreen1-hNSCs expressed GFAP (36.8 ± 7.4%, P < 0.0001) (**D**). Thus, Zfp488 expression largely prevented an astrocyte fate after differentiation. Scale-50 μM.

After culturing the H9 derived NSCs for three weeks in OPC differentiation medium, 53.5 ± 7.2% of control ZsGreen1-hNSCs expressed the astrocyte marker S100 beta (Fig. 2B). In contrast, a significantly lower fraction of Zfp488-ZsGreen1-hNSCs expressed S100 beta (6.8 ± 3.5%, P < 0.001) (Fig. 2A). Cells were further allowed to differentiate up to 6 weeks to allow for GFAP expression. A lower fraction (10.6 ± 1.4%) of Zfp488-ZsGreen1-hNSC expressed GFAP (Fig. 2E), while a significantly large fraction (36.8 ± 7.4%, P < 0.0001compared to Zfp488 group) of control ZsGreen1-hNSC expressed GFAP (Fig. 2D). Bar graph (Fig. 2C) shows the percentages of S100beta^+^/ZsGreen1^+^ and GFAP^+^/ZsGreen1^+^ cells. Thus, Zfp488 expression largely created an oligodendrocyte lineage bias during differentiation of the hNSCs.

### ZFP488 misexpression increases the yield of hNSC–derived OPCs, and mature oligodendrocyte *in vitro*

**Figure 3:**
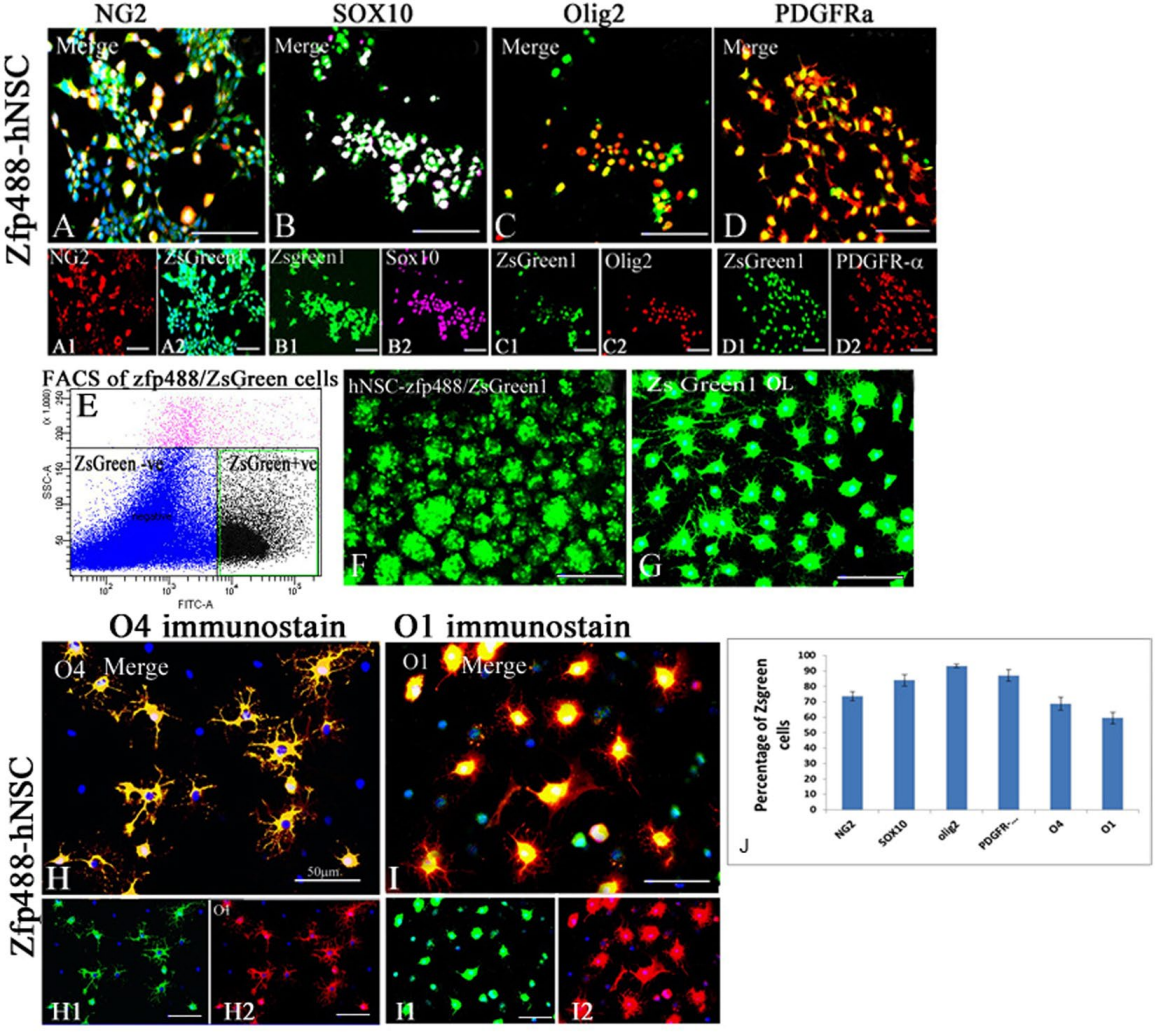
For directed OPC differentiation the Zfp488-ZsGreen1-hNSCs were plated as a monolayer on laminin-coated plates. In Zfp488 group, after 4–5 weeks of differentiation 74.0 ± 3.0% of ZsGreen1 positive cells were NG2^+^ (**A**-**A2**), 83.8 ± 3.8% cells were sox10^+^ (**B**-**B2**), 93.2 ± 1.2%, Olig2^+^ (**C**-**C2**) and 87.0 ± 4.2% PDGFR-alpha^+^ (**D**-**D2**). The bar graph shows the percentages of green cells that express different markers (**J**). We could not derive quantifiable cells expressing OPC markers from control hNSCs using this protocol. Next, the H9-derived Zfp488 NSCs were FAC sorted using ZsGreen1 fluorescence reporter using FITC-A channel on Aria II FACS (**E**). The sorted cells formed spheres after overnight culture in ultralow attachment plates (**F**) and could be easily expanded in neural expansion media and formed a monolayer after plating on laminin-coated plates (**G**). FACS sorted ZsGreen1-OPCs, were induced to further differentiation. The percentage of Zfp488 cells expressing O4 was 68.7 ± 4.2% at day 14 after differentiation with thyroid hormone and morphologically resembled mature oligodendrocytes (**H**-**H2**). Further differentiation for at least 1 more week resulted in approximately 59.5 ± 3.7% of ZsGreen1^+^/O1^+^ cells (**I**-**I2**).

The percentages of ZsGreen1 cells expressing stage-specific OPC markers were evaluated by immunohistochemistry (Fig. 3). The cells that co-labeled for ZsGreen1 and various OPC markers were counted and expressed as a percentage of total ZsGreen1^+^ cells. In Zfp488 group, after 4–5 weeks of differentiation 74.0 ± 3.0% of ZsGreen1 cells were NG2^+^ (Fig. 3A), 83.8 + 3.8% cells were sox10^+^ (Fig. 3B), 93.2 + 1.2% were Olig2^+^ (Fig. 3C) and 87.0 + 4.2% were PDGFR-α^+^ (Fig. 3D). The bar graph shows the percentages of green cells that express different markers (Fig. 3J). We could not derive quantifiable cells expressing OPC markers from control hNSCs using this protocol (less than 5% NG2 positive cells were seen after 4 weeks but they could not survive for more than a week).

Next, the H9-derived Zfp488 NSCs were FAC sorted using ZsGreen1 fluorescence (BD FACsAria II and BD FACSDiva software, USA) (Fig. 3E). The sorted cells formed spheres after overnight culture in ultralow attachment plates and could be easily expanded in neural expansion media (Fig. 3F). After plating the spheres, cells formed a monolayer within a few days with a mean doubling time of 48 hours (Fig. 3G). Since Zfp488 is a critical TF expressed during differentiation of OPCs during development, we took FACS sorted ZsGreen1^+^/OPCs and induced them to further differentiate. The percentage of Zfp488^+^ cells expressing the pre-OL marker O4 was 68.7 ± 4.2% at day 14 after differentiation with thyroid hormone (Fig. 3H). Further differentiation for at least 1 additional week resulted in approximately 59.5 ± 3.7% of ZsGreen1^+^ were O1^+^ (Fig. 3I). We detected a few ZsGreen1 positive astrocytes (not shown). Therefore, Zfp488 misexpression allowed the terminal differentiation to mature OLs after directed invitro differentiation. Thus, ZFP488 expression alone was enough to induce expression of markers of the OPC/oligodendrocyte lineage in differentiating NSC that were generated without dorso-ventral patterning of EB with morphogens.

### Expression of Zfp488 during spontaneous differentiation of H9 derived hNSCs prevented their default neuronal fate ***in vitro***

Following 3 weeks of spontaneous differentiation of monolayer cultures, majority of the untransduced (supplementary documents Fig. 1A) and control ZsGreen1-hNSCs (green cells, Supplementary Fig. 1B) differentiated into beta III tubulin positive immature neurons. In contrast, Zfp488^+^/ZsGreen1^+^ hNSCs cells rarely differentiated into beta-tubulin positive neurons (Supplementary Fig. 1C). Thus, expression of Zfp488 prevented the hNSC from acquiring their default fate of differentiating into neurons after removal of growth factors. Similar results were obtained from Zfp488 expressing neurospheres (Supplementary Fig. 2).

### Migration of transplanted H9 hESC derived Zfp488-ZsGreen1-hNSCs and control-NSCs in a model of focal demyelination model

**Figure 4:**
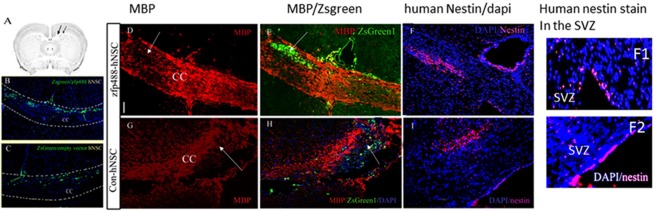
Transplantation of Zfp488-ZsGreen1-hNSCs and control ZsGreen1-hNSCs in the corpus callosum (CC) of mice near a demyelinating lesion for *in vivo* differentiation. (**A**) Schematic diagram of the NSC injection site and lysolecithin injection site in the CC. Transplanted (ZsGreen1 reporter) cells in both groups were mainly distributed along the corpus callosum (low power image, **B**,**C**). A single dose of lysolecithin resulted in focal demyelination at the injection site as indicated by a loss in MBP staining (**D**,**G**). Both Zfp488-ZsGreen1-hNSCs and control ZsGreen1-hNSCs migrated towards the lesion site (**E**,**H** respectively). The transplanted human NSCs were also identified by an antibody specifically against human nestin (**F**,**I**). A small fraction of human nestin-positive cells were detected in the SVZ from both groups, indicating possible homing of hNSCs (F1-F2). The scale is 50 μM.

We assessed the fate of zf488-ZsGreen1-hNSCs *in vivo* in a context-dependent manner by transplanting them near the site of an acute demyelinating lesion in the corpus callosum (Fig. 4A–C). Control ZsGreen1-hNSCs (carrying only the reporter) were also transplanted under the same experimental conditions in different mice. A single injection of lysolecithin in the corpus callosum of adult mice resulted in focal demyelination within 48–72 hours (arrows, Fig. 4D,G) that typically elicits a repair response leading to spontaneous remyelination beginning within 3–4 weeks. This provided an environment where host-derived cues required for NSC migration and differentiation, and OPC maturation were present immediately after transplantation.

A fraction of Zfp488-ZsGreen1-hNSCs and control hNSCs were engrafted as detected by ZsGreen1 reporter positive cells (at or near the injection site) and survived for at least 4 weeks (Fig. 4B,C). Both hNSCs migrated along the corpus callosum towards the lesion site and populated the area in and around it (Fig. 4E,H). NSCs were also detected by human-specific nestin antibody. Human nestin^+^ NSCs were found mostly concentrated at the lesion site in the corpus callosum (Fig. 4F,I). Although the injection site was far from SVZ, the presence of exogenous NSC was detected with human nestin antibody in SVZ in both groups (Fig. 4F1-2). Since the NSCs remained undifferentiated hence no ZsGreen1 fluorescence was detected.

### Transplanted Zfp488-hNSC differentiates efficiently into OPCs and OLs than control hNSC under a demyelinating condition

**Figure 5:**
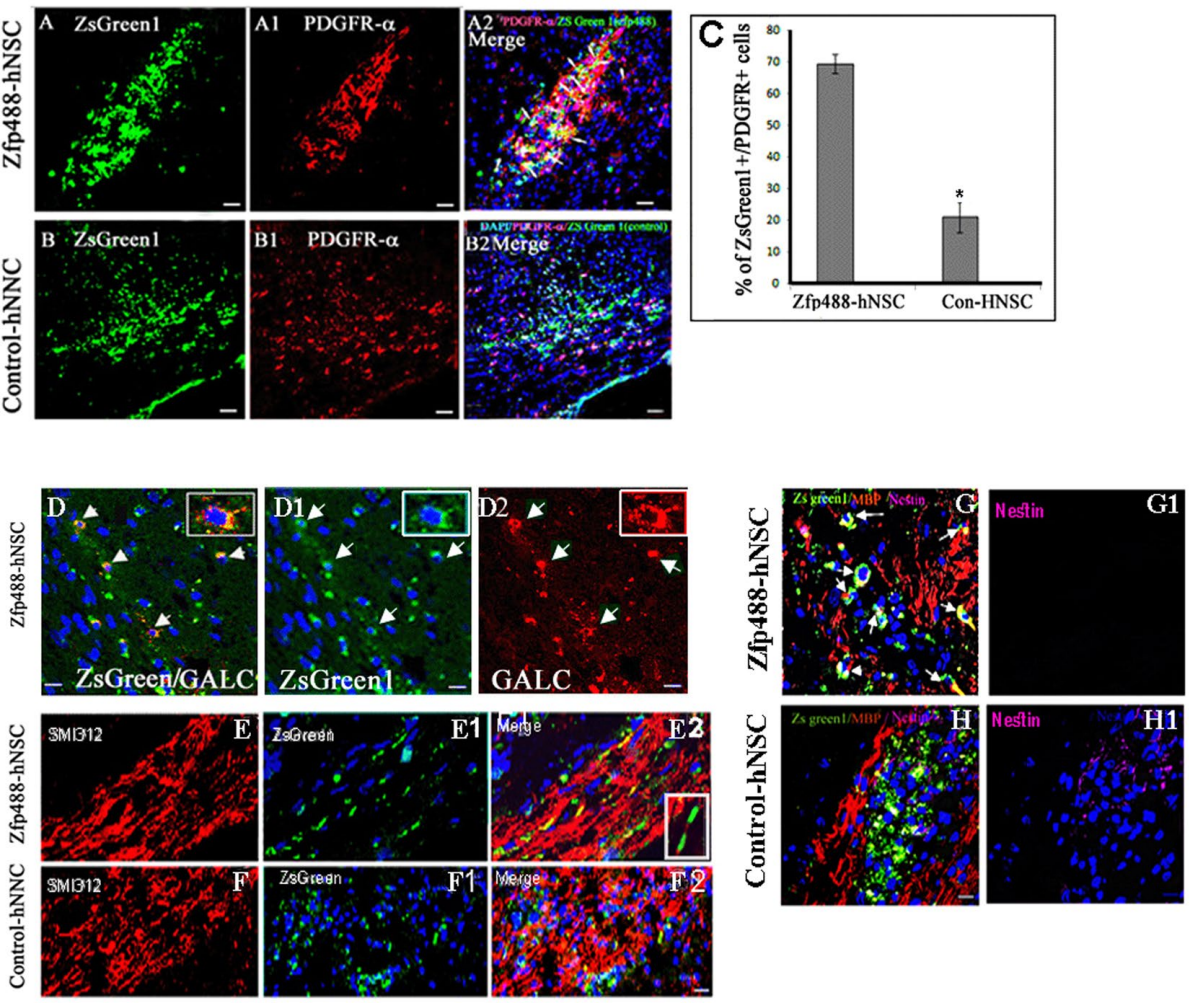
Comparison of *in vivo* differentiation of Zfp488-ZsGreen1-hNSCs and control-hNSCs into PDGFR-alpha positive OPCs within the lesion in the CC (**A**,**B** respectively). In Zfp488 group, 69.4 ± 3.0% of ZsGreen1 cells coexpressed PDGFR-α (see arrows in A2). The PDGFR-α^+^ OPCs exhibited a relatively more mature morphology than the control group as evident by their extended processes. In contrast, 20 ± 4.7% of the control-hNSCs were found to differentiate into PDGFR-α expressing OPCs as shown by co-labeling of PDGR-α and ZsGreen1 (**B**-**B2**). (**C**) Quantification of PDGFR-α immunohistochemistry. A few ZsGreen1^+^/GALC^+^ double labeled were also seen in the Zfp488-ZsGreen1-hNSC group (5D-D2). Confocal images of neurofilament immunostaining (with SM312 antibody) showed that ZsGreen1^+^ tracts coaligned with the neurofilament tracts in Zfp488-ZsGreen1-hNSC transplanted brains (**E**-**E2**). Inset (**E2**) shows the wrapping of ZsGreen1 positive sheath around a section of SMI312^+^ axon. No co-labeling was seen with control hNSCs (**F**-**F2**). Within the lesion, in the Zfp488 group, ZsGreen1^+^/MBP^+^/nestin^−^ cells were detected (**G**-**G1**, arrows). In contrast, in control NSC group, many ZsGreen1 cells were nestin positive indicating that they did not differentiate (**H**-**H1**). Blue nuclei, Hoechst staining. Bar (for all panels) is equal to 100 μM. Abbreviations: CC, corpus callosum; GFAP, glial fibrillary acidic protein; ZsGreen1, Zoanthus sp green fluorescent protein.

Immunofluorescence staining using antibodies against OPC lineage markers showed that 69.4 ± 3.0% of ZsGreen1^+^ cell in Zfp488-ZsGreen1-hNSCs coexpressed PDGFR-α (Fig. 5A-A2,C) compared to 20.0 ± 4.7% (P < 0.01) in control group (Fig. 5B-B2,C). The PDGFR-α^+^ OPCs were also of more mature morphology than the control group as evident by their long processes (Fig. 5A1). We found that 10.0 ± 0.8% of ZsGreen1^+^/Zfp488-ZsGreen1-hNSCs cells coexpressed the GALC, a marker for mature oligodendrocytes (Fig. 5D-D2). We did not detect ZsGreen1/GALC positive cells in the control hNSC group (not shown). In the Zfp488 group, we detected ZsGreen1^+^ tracks overlapped with the SMI312^+^ axonal tracts (Fig. 5E-E2). We did not detect similar tracks in the control group (Fig. 5F-F2). In the Zfp488 group, we also detected a few ZsGreen1^+^ cells expressing MBP, and nestin was not expressed by any green cells. In contrast, in the control group, many ZsGreen1 cells still expressed nestin indicating that they remained immature (Fig. 5G-G1,H-H1 respectively). We also did not detect any ZsGreen1^+^ cells expressing neuronal marker Tuji in any group (not shown). Thus, Zfp488-ZsGreen1-hNSCs predominantly differentiated into oligodendrocyte lineage cells within 4 weeks *in vivo*.

### Transplanted Zfp488-hNSC rarely differentiated into astrocytes at the lesion site

**Figure 6:**
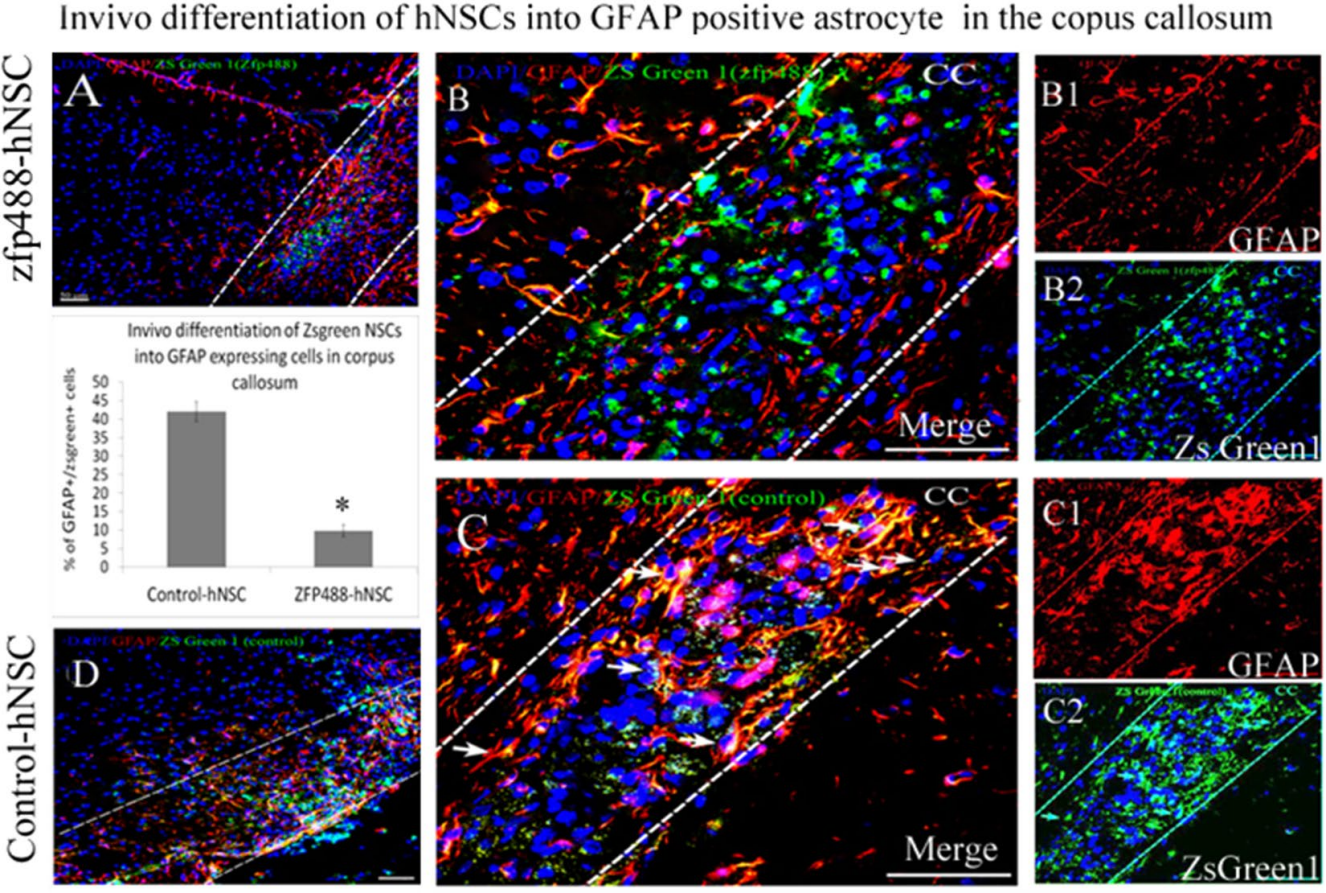
Comparison of *in vivo* differentiation of Zfp488-ZsGreen1-hNSCs and control-hNSCs into astrocytes within the demyelinating lesion in the CC using GFAP antibody (**A**,**D**, low power). Co-labeled GFAP^+^ (red) (**B1**), and ZsGreen1^+^ cells (**B2**) revealed that only a few double-labeled cells (**B**, arrows) among the transplanted surviving Zfp488-ZsGreen1-hNSCs had differentiated into an astrocytic cell lineage. In contrast, numerous transplanted control ZsGreen1-hNSCs were found to differentiate into GFAP^+^ astrocytes (red, fig. **C**, arrows), as shown by co-labeling of GFAP (red, **C1**), and ZsGreen1 reporter (**C2**). Quantitative analysis of the confocal images showed that 9.8 ± 2.6% in Zfp488-hNSCs differentiated into GFAP astrocytes while 42.0 ± 2.2% in control-hNSCs differentiated into GFAP^+^ astrocytes (see bar graph). Blue nuclei, Hoechst staining. Bar (for all panels) is equal to 100 μM. Abbreviations: CC, corpus callosum; GFAP, glial fibrillary acidic protein; ZsGreen1, Zoanthus sp green fluorescent protein.

9.8 ± 2.6% of Zfp488-ZsGreen1-hNSCs co-labeled with GFAP at the lesion site indicating a low potential for differentiation into astrocytes (Fig. 6A,B,B2). In contrast, 42.0 ± 2.2% of Zsgreen^+^ control-hNSCs showed astroglial differentiation predominantly in the lesion area as evidenced by significantly greater (P < 0.001) co-labeling of ZsGreen1 and GFAP signals (analyzed using a confocal microscope) (Fig. 6D,C-C2). In both groups of mice, endogenous astrogliosis was evidenced by the presence of GFAP^+^/ZsGreen1^−^ cells seen at the lesion site. The bar graph shows quantification of immunohistochemistry data.

### Zfp488-OLs can myelinate rat dorsal root ganglion neurons *in vitro* co-cultures

**Figure 7:**
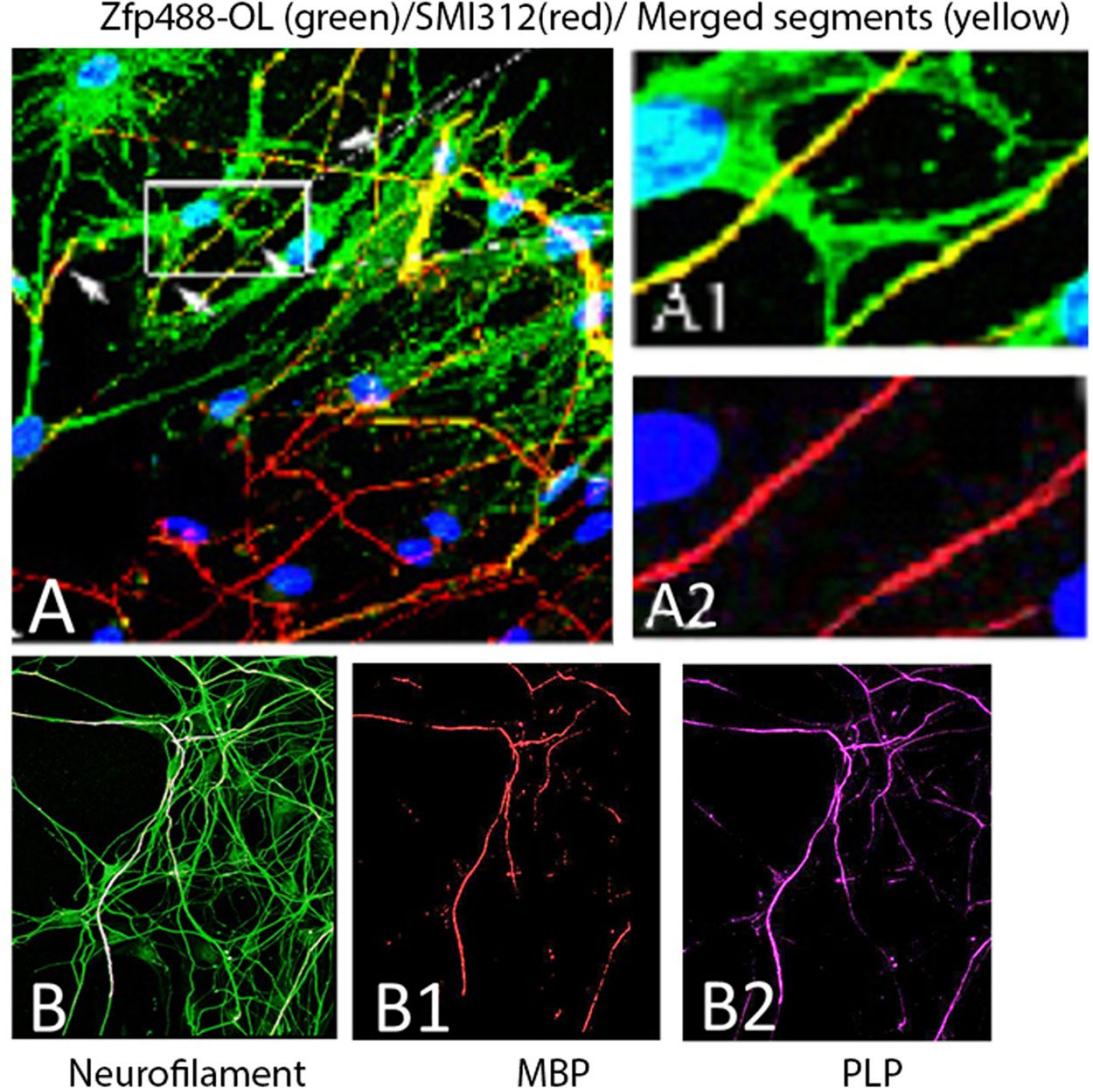
Zfp488-ZsGreen1-hNSC derived OLs can myelinate rat dorsal root ganglion neurons (DRG) *in vitro* (**A**-**A2** and **B**-**B2**). Confocal images of the co-culture between rat DRG neurons and iPSC derived Zfp488-ZsGreen1-OLs showing multiple ZsGreen1^+^ OLs in contact with SMI312^+^ (red) axons. The co-labeled areas appear yellow. (**A1**,**A2**) Magnification of a representative region with a mature OL extending its processes over axons stained with SMI312 (red, **A2**). The co-labeled areas appear yellow. (**B**) Sections of SMI312^+^ (green) axons were wrapped with MBP^+^ (red, **B1**) and PLP^+^ (magenta, **B2**) myelin sheath. Scale bars: 10 μM (**A**-**A2**), 50 μM (**B**-**B2**) 10 μM.

Zfp488-ZsGreen1-hNSCs derived O4 expressing OLs were plated on top of the 15 days old DRG neurons (Lonza, USA) and were co-cultured for three weeks in OL myelination medium (see supplementary document 1 for information on media). By two weeks of co-culture, extensions of Zfp488-ZsGreen1-OLs (identified by green cell body and processes) aligned with the pan axonal marker SMI312^+^ axons at multiple locations (Fig. 7A-A2). Confocal images show the presence of ZsGreen1^+^ (green) sheaths wrapped around more than one axon (Fig. 7A1). By 3 more weeks, large segments of SMI312^+^ axons (green) were wrapped by with MBP and PLP positive myelin sheath (Fig. 7B-B2). ZsGreen1 expression was extremely low at this point in the Zfp488 oligodendrocytes.

### Human iPSCs derived Zfp488-NSCs differentiated into OPCs that generated functional OLs that formed multilayered myelin in myelin deficient shiverer mouse brain

**Figure 8:**
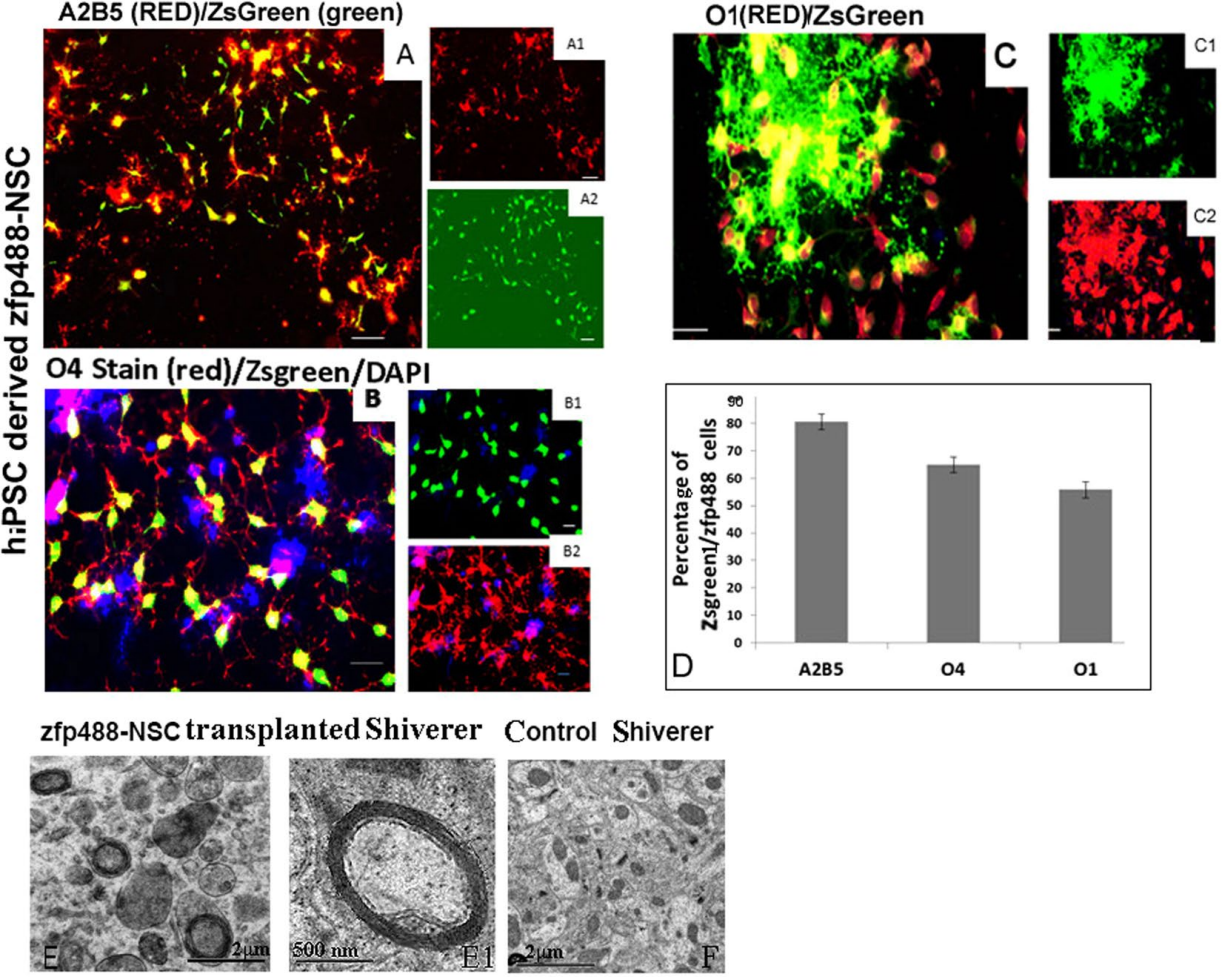
Zfp488-NSCs derived from iPSCs, differentiated into functional oligodendrocytes that generated multilayer myelin sheath in Shiverer mice. (**A**) ZsGreen1 expressing cells co-expressed A2B5 (**A1**,**A2** are individual channels). (**B**) ZsGreen1 cells co-expressed O4 (**B1**,**B2** are individual channels). (**C**) ZsGreen1 cells co-expressed O4 (**C1**,**C2** are individual channels). (**D**) The percentages of ZsGreen1 cells that were positive for A2B5 were 80.5 ± 2.7 (**A**), for O4 64.8 ± 2.8 (**B**), and for O1 55.8 ± 2.9 (**D**). (**E**) Zfp488-NSC derived O4^+^ OPCs were transplanted in the corpus callosum of adult shiverer mice. Under the electron microscope, multilayered myelin sheaths were visible around axons (**E**,**E1**). (**F**) Corpus callosum of control untransplanted Shiverer mouse lacked multilayered compact myelin sheath at a similar age (**F**). The bar is 500 nM.

To demonstrate the robustness of the protocol, we also generated Zfp488-NSCs derived OLs from a human iPSCs. The percentages of ZsGreen1 cells that were A2B5^+^ (after three weeks in OPC 2 media) was 80.5 ± 2.7% (Fig. 8A-A2). After four weeks of differentiation, the percentage of ZsGreen1 cells that were PDGFR-α^+^ was 80.5 ± 2.7% (Supplementary Fig. 4). The percentage of O4^+^ cells was 64.8 ± 2.8% after 7–8 weeks of differentiation (Fig. 8B-B2), and 55.8 ± 2.9% were O1^+^ (Fig. 8D-D2). Control NSCs primarily differentiated into Tuji positive neurons (~78%), S100-beta^+^ astrocytes (~27%), and few O4^+^ cells which did not divide (~8%)(Supplementary Fig. 5). To demonstrate that Zfp488-ZsGreen1-hNSC derived OLs can form multilayered myelin, we transplanted O4^+^ cells in the corpus callosum of adult shiverer mouse (N = 3). Mice were sacrificed after 8 weeks and the brain was processed for EM. Zfp488 expressing OLs successfully myelinated the axons in shiverer mice. Multilayered compact myelin sheath was seen around several axons (Fig. 8E-E1). We did not detect multilayered compact myelin in the corpus callosum of control un-transplanted shiverer mice (Fig. 8F).

## Discussion

A key prerequisite for the biomedical applications of human NSCs for oligodendrocyte replacement is their controlled differentiation into oligodendrocyte lineages cells *in vivo*. The zinc-finger protein Zfp488 is an oligodendrocyte-specific critical transcriptional co-regulator. It is expressed in newly formed OLs and again during terminal differentiation of OL in rodents and human. Specifically, Zfp488 physically interacts with another OL lineage-specific transcription factor Olig2 to promote oligodendroctye differentiation^33^. In this study, we show that forced expression of Zfp488 in human NSCs (hESC or iPSC derived) during their differentiation directs them predominantly towards an oligodendrocyte fate both *in vitro* and *in vivo*. For comparing the oligodendrocyte fate of the transplanted NSCs *in vivo*, we chose a limited time frame of four weeks since majority of control-hNSCs remain undifferentiated or differentiate into astrocytes during that time^42^. We found that within four weeks of transplantation into the mouse corpus callosum the majority of Zfp488 expressing hNSCs appeared to have differentiated into OPCs or premyelinating oligodendrocytes (with nominal astrocyte differentiation).

Interestingly, both rodent and human NSCs generally show a clear bias toward differentiation into astroglia *in vivo*^42,43^. Specifically, hESC derived hNSCs primarily give rise to astrocytes (35–60%) and nominal oligodendrocytes (2–5%) *in vitro*^44–46^ and also after transplantation in the demyelinating models. However, primary human NSCs artificially overexpressing the olig2 transcription factor gene can differentiate predominantly into myelinating oligodendrocytes and contribute to the remyelination of the corpus callosum as well as promote locomotor recovery^30^. Overexpression of Olig2 has been reported to be associated with specific aspects Down syndrome pathology in the brain, therefore the significance of olig2 overexpression in NSC remains to be studied. Our focus was on Zfp488 since studies from our own laboratory have shown that mouse SVZ NSCs when transduced *in situ* with Zfp488-retrovirus, specifically adopted an oligodendrocyte fate after differentiation following cuprizone-induced demyelination^32^. Moreover, compared to olig2 and sox10^47–50^, Zfp488 is exclusively expressed in oligodendrocyte lineage cells^33^. Here we show that human Zfp488-hNSC also acquire similar fate *in vivo* in response to demyelination/remyelination signals.

One of the objectives of this study was to compare the *in vitro* differentiation fate of Zfp488-hNSC compared to naïve hNSC. As expected, hNSC whether naive or transduced with empty vector, primarily generated beta-tubulin positive immature neuron, followed by S100beta and GFAP expressing astrocytes, but rarely oligodendrocytes. Thus, this condition was ideal as a suitable baseline for assessing the potential pro-oligodendrogliogenic effects of Zfp488. We found that Zfp488 expression inhibited neuronal fate completely while generating OPCs, clearly with an oligodendrocyte bias as seen by significantly higher O4-positive oligodendrocytes. Furthermore, pre-OLs emerging from Zfp488-hNSC cultures showed a mature phenotype with highly branched processes and formation of membrane sheet extensions. Overall, our data support the view that Zfp488 expression efficiently directs the fate of NSC towards oligodendrocyte fate.

Our second objective was to compare the *in vivo* differentiation phenotypes of Zfp488-ZsGreen1-hNSCs with control hNSCs. The four-week time point was chosen since numerous previous studies have reported that hESC derived NSCs remained mostly undifferentiated when transplanted in demyelination models when detected after 3–4 weeks^30,51^. We demonstrated the ability of Zfp488-ZsGreen1-hNSCs to respond appropriately to the remyelinating cues *in vivo*, which include migrating towards the LPC lesion, and differentiate into OPC in a demyelinating-remyelinating environment. Although there are many approaches for testing these qualities, we used LPC induced demyelination model to assess their fate in a system favoring NSC differentiation without any significant inflammation. Following transplantation, the control hNSCs migrated to the lesion area, where they differentiated predominantly into GFAP expressing astrocytes, while rest of the cells remained nestin-positive undifferentiated cells. Similar to other reports, we failed to detect any transplanted control hNSC derived OPC or mature oligodendrocytes confirming the low oligodendroglial differentiation efficiency of human NSCs compared with their rodent counterparts^52^. In contrast, Zfp488 positive cells expressed the OPC marker PDGFRα, and astrocyte marker GFAP. Neuronal differentiation was not detected in our system for any group, primarily since the corpus callosum is a white matter tract (nonneuronogenic area). Additionally, Zfp488 hNSCs differentiated into late OPC marker O4 expressing OLs and mature premyelinating OLs expressing MBP, O1, and GALC. We identified ZsGreen1 (Zfp488) positive tract parallel track in the corpus callosum which colocalized with MBP and neurofilament staining displaying evidence of initiation of myelinating by transplanting cells. Thus, misexpression of Zfp488 resulted in efficient differentiation of OPC into mature OL than naïve OPCs. We also detected human nestin-positive NSCs in the SVZ area indicating that the Zfp488-NSCs were capable of migrating to the niche area.

To evaluate the myelination capacity of the OPCs derived from Zfp488-NSCs, we injected them into the corpus callosum of the shiverer mouse brain. The Zfp488 hNSCs could differentiate *in vivo* in the myelin-deficient shiverer mice and successfully generated myelin sheath around the naked axons. In addition, the myelinating capacity of iPSC derived Zfp488-NSCs was demonstrated in rat DRG–OPC co-culture. These data indicate that direct misexpression of Zfp488 can be used to produce lineage-restricted hNSCs with a high capacity for OPC generation and finally functional oligodendrocytes.

The effects of Zfp488 misexpression in this study could be due to a general shift in lineage differentiation and an acceleration of oligodendrocyte maturation. Recently, by combining chromatin immunoprecipitation sequencing (ChIP-seq) and RNA sequencing (RNA-seq) data, Znf488 (the human ortholog of mouse Zfp488) was identified as a critical patterning transcription factor in the ventral spinal cord involved in defining the P1 and P2 domain progenitor identity in the chicken^53^. It was shown that Znf488 was expressed together with olig2 in the P2/pre-motor neuron (pMN) domain at stage HH14 of the chicken neural tube. The pMN domain gives rise to OPCs following motor neuron generation. It is possible that mouse ZFP488 may also serve as a patterning regulator and a morphogen capable of changing the lineage of progenitors towards an oligodendroglial fate. Overall, it could be speculated that a novel role of Zfp488 as a neural progenitor patterning factor may explain why a TF required for OPC formation and maturation can regulate the fate of NSCs towards oligodendrocytes.

An alternate mechanism for the robust oligodendrocyte fate of the Zfp488-NSCs could be as associated maintenance of sox9 expression. The transcription factor Sox9 strongly influences the multipotential neural stem cells toward an OPC fate^54^. Oligodendrocyte specification via SOXE expressions in the CNS is temporally orchestrated starting with sox9 expression in the spinal cord neuroepithelium, persisting in OPCs and maintained in adult astrocytes^54^, followed by Sox8 and then sox10 expression. As presented in Supplementary Fig. 6, sox9 mRNA levels were reduced when control NSCs were differentiated for 3 days in OPC media, whereas the levels remained unchanged in Zfp488-NSCs under the same differentiating conditions. Sox9 mRNA levels were similar between undifferentiated Zfp488-NSC and control-NSCs since Zfp488 expression was not switched on before differentiation (discussed in the introduction). Similarly, after transplantation of the control and Zfp488-NSCs in the LPC mice model, we detected a significantly higher percentage of ZsGreen1 cells that were Sox9 positive in the Zfp488 group, compared to control ZsGreen1 cells, indicating maintenance of sox9 expression in Zfp488 expressing cells (Supplementary Fig. 6). It has been reported that knocking out Sox9 results in reduced oligodendrocytes and increased numbers of motor neurons generated by neural progenitors in the ventral spinal cords of mice^45^. Additional studies show that neurospheres infected with sox9 or sox10 under OL differentiating conditions show a significant increase in OPC generation, demonstrating that that SOX9 and SOX10 enhance OPC specification and maturation^55^. Sox9 is however expressed earlier than sox10 during development. The effect on Sox9 on Zfp488 mediate OPC fate restriction could be either direct or indirect. Overall, our protocol used for neural differentiation of human ES/iPSC derived neural stem cells, allows for large scale production of uniform, Zfp488-ZsGreen1-hNSCs with a predominant oligodendroglial lineage differentiation potential.

## Supplementary information

Supplementary information

## References

[1] Weiss S (1996). Multipotent CNS stem cells are present in the adult mammalian spinal cord and ventricular neuroaxis. The Journal of neuroscience: the official journal of the Society for Neuroscience.

[2] McKay R (1997). Stem Cells in the Central Nervous System. Science (New York, N.Y.).

[3] Reynolds BA, Weiss S (1992). Generation of neurons and astrocytes from isolated cells of the adult mammalian central nervous system. Science (New York, N.Y.).

[4] Flax JD (1998). Engraftable human neural stem cells respond to developmental cues, replace neurons, and express foreign genes. Nature biotechnology.

[5] Pluchino S, Martino G (2008). The therapeutic plasticity of neural stem/precursor cells in multiple sclerosis. Journal of the neurological sciences.

[6] Aharonowiz M (2008). Neuroprotective effect of transplanted human embryonic stem cell-derived neural precursors in an animal model of multiple sclerosis. PloS one.

[7] Imitola J (2004). Directed migration of neural stem cells to sites of CNS injury by the stromal cell-derived factor 1alpha/CXC chemokine receptor 4 pathway. Proceedings of the National Academy of Sciences of the United States of America.

[8] Mothe AJ, Tator CH (2005). Proliferation, migration, and differentiation of endogenous ependymal region stem/progenitor cells following minimal spinal cord injury in the adult rat. Neuroscience.

[9] Ke Y (2006). Early response of endogenous adult neural progenitor cells to acute spinal cord injury in mice. Stem cells (Dayton, Ohio).

[10] Kernie SG, Parent JM (2010). Forebrain neurogenesis after focal Ischemic and traumatic brain injury. Neurobiology of disease.

[11] Capilla-Gonzalez V (2014). The subventricular zone is able to respond to a demyelinating lesion after localized radiation. Stem cells (Dayton, Ohio).

[12] Nait-Oumesmar B (1999). Progenitor cells of the adult mouse subventricular zone proliferate, migrate and differentiate into oligodendrocytes after demyelination. The European journal of neuroscience.

[13] Nait-Oumesmar B, Picard-Riera N, Kerninon C, Baron-Van Evercooren A (2008). The role of SVZ-derived neural precursors in demyelinating diseases: from animal models to multiple sclerosis. Journal of the neurological sciences.

[14] Romanko MJ (2004). Roles of the mammalian subventricular zone in cell replacement after brain injury. Progress in neurobiology.

[15] Nishino H, Borlongan CV (2000). Restoration of function by neural transplantation in the ischemic brain. Progress in brain research.

[16] Buchet D, Baron-Van Evercooren A (2009). In search of human oligodendroglia for myelin repair. Neuroscience letters.

[17] Pluchino S (2009). Human neural stem cells ameliorate autoimmune encephalomyelitis in non-human primates. Annals of Neurology.

[18] Darsalia V, Kallur T, Kokaia Z (2007). Survival, migration and neuronal differentiation of human fetal striatal and cortical neural stem cells grafted in stroke-damaged rat striatum. The European journal of neuroscience.

[19] Lakatos A, Franklin RJ (2002). Transplant mediated repair of the central nervous system: an imminent solution?. Current opinion in neurology.

[20] Gowing G (2014). Glial cell line-derived neurotrophic factor-secreting human neural progenitors show long-term survival, maturation into astrocytes, and no tumor formation following transplantation into the spinal cord of immunocompromised rats. Neuroreport.

[21] Das MM (2016). Human neural progenitors differentiate into astrocytes and protect motor neurons in aging rats. Experimental Neurology.

[22] Rao MS, Mayer-Proschel M (2000). Precursor cells for transplantation. Progress in brain research.

[23] McBride JL (2004). Human neural stem cell transplants improve motor function in a rat model of Huntington’s disease. The Journal of comparative neurology.

[24] Tarasenko YI (2007). Human fetal neural stem cells grafted into contusion-injured rat spinal cords improve behavior. Journal of neuroscience research.

[25] Cummings BJ (2005). Human neural stem cells differentiate and promote locomotor recovery in spinal cord-injured mice. Proceedings of the National Academy of Sciences of the United States of America.

[26] Ben-Hur T (2007). Serial ***in vivo*** MR tracking of magnetically labeled neural spheres transplanted in chronic EAE mice. Magnetic resonance in medicine: official journal of the Society of Magnetic Resonance in Medicine / Society of Magnetic Resonance in Medicine.

[27] Giannakopoulou A (2011). Inflammatory changes induced by transplanted neural precursor cells in a multiple sclerosis model. Neuroreport.

[28] Pluchino S (2005). Neurosphere-derived multipotent precursors promote neuroprotection by an immunomodulatory mechanism. Nature.

[29] Gao Z (2014). Osthole augments therapeutic efficiency of neural stem cells-based therapy in experimental autoimmune encephalomyelitis. Journal of pharmacological sciences.

[30] Sher F (2012). Intraventricularly injected Olig2-NSCs attenuate established relapsing-remitting EAE in mice. Cell transplantation.

[31] Hofstetter CP (2005). Allodynia limits the usefulness of intraspinal neural stem cell grafts; directed differentiation improves outcome. Nature Neuroscience.

[32] Soundarapandian MM (2011). Zfp488 promotes oligodendrocyte differentiation of neural progenitor cells in adult mice after demyelination. Scientific reports.

[33] Wang SZ (2006). An oligodendrocyte-specific zinc-finger transcription regulator cooperates with Olig2 to promote oligodendrocyte differentiation. Development (Cambridge, England).

[34] Zhang Y (2014). An RNA-sequencing transcriptome and splicing database of glia, neurons, and vascular cells of the cerebral cortex. The Journal of neuroscience: the official journal of the Society for Neuroscience.

[35] Thomson JA (1998). Embryonic stem cell lines derived from human blastocysts. Science (New York, N.Y.).

[36] Yu J (2007). Induced pluripotent stem cell lines derived from human somatic cells. Science (New York, N.Y.).

[37] Mitalipova M (2003). Human embryonic stem cell lines derived from discarded embryos. Stem cells (Dayton, Ohio).

[38] Shinkai Y (1992). RAG-2-deficient mice lack mature lymphocytes owing to inability to initiate V(D)J rearrangement. Cell.

[39] Chernoff GF (1981). Shiverer: an autosomal recessive mutant mouse with myelin deficiency. The Journal of heredity.

[40] Matthieu JM, Tosic M, Roach A (1992). Myelin-deficient mutant mice. An *in vivo* model for inhibition of gene expression by natural antisense RNA. Annals of the New York Academy of Sciences.

[41] Dodge JE, Ramsahoye BH, Wo ZG, Okano M, Li E (2002). De novo methylation of MMLV provirus in embryonic stem cells: CpG versus non-CpG methylation. Gene.

[42] Cao QL (2001). Pluripotent stem cells engrafted into the normal or lesioned adult rat spinal cord are restricted to a glial lineage. Experimental Neurology.

[43] Itakura G (2015). Controlling Immune Rejection Is a Fail-Safe System against Potential Tumorigenicity after Human iPSC-Derived Neural Stem Cell Transplantation. PloS one.

[44] Vescovi AL, Gritti A, Galli R, Parati EA (1999). Isolation and intracerebral grafting of nontransformed multipotential embryonic human CNS stem cells. Journal of Neurotrauma.

[45] Caldwell MA (2001). Growth factors regulate the survival and fate of cells derived from human neurospheres. Nature biotechnology.

[46] Cavazzin C (2006). Unique expression, and localization of aquaporin-4 and aquaporin-9 in murine and human neural stem cells and in their glial progeny. Glia.

[47] Zhou Q, Anderson DJ (2002). The bHLH transcription factors OLIG2 and OLIG1 couple neuronal and glial subtype specification. Cell.

[48] Masahiro N (2006). Olig2-positive progenitors in the embryonic spinal cord give rise not only to motoneurons and oligodendrocytes but also to a subset of astrocytes and ependymal cells. Developmental biology.

[49] Aoki Y (2003). Sox10 regulates the development of neural crest-derived melanocytes in Xenopus. Developmental biology.

[50] McKeown SJ, Lee VM, Bronner-Fraser M, Newgreen DF, Farlie PG (2005). Sox10 overexpression induces neural crest-like cells from all dorsoventral levels of the neural tube but inhibits differentiation. Developmental dynamics: an official publication of the American Association of Anatomists.

[51] Copray S, Huynh JL, Sher F, Casaccia-Bonnefil P, Boddeke E (2009). Epigenetic mechanisms facilitating oligodendrocyte development, maturation, and aging. Glia.

[52] Svendsen CN, Caldwell MA, Ostenfeld T (1999). Human neural stem cells: isolation, expansion, and transplantation. Brain pathology (Zurich, Switzerland).

[53] Rahimi R (2016). Epigenomics-Based Identification of Major Cell Identity Regulators within Heterogeneous Cell Populations. Cell reports.

[54] Stolt CC (2003). The Sox9 transcription factor determines glial fate choice in the. Genes & development.

[55] Pozniak CD (2010). Sox10 directs neural stem cells toward the oligodendrocyte lineage by decreasing Suppressor of Fused expression. Proceedings of the National Academy of Sciences of the United States of America.

