# Supplementary material for: Development of glial restricted human neural stem cells for oligodendrocyte differentiation in vitro and in vivo
Source: Sci Rep. 2019 Jun 21;9:9013. doi: 10.1038/s41598-019-45247-3 (PMC6588721; doi:10.1038/s41598-019-45247-3)
Supplement: Supplementary file 1 — Supplementary information [file 41598_2019_45247_MOESM1_ESM.pdf]

Supplementary Information

Development of glial restricted human neural stem cells for oligodendrocyte differentiation in vitro and in vivo

Sangita Biswas, Seung Hyuk Chung, Peng Jiang, Samaneh Dehghan, Wenbin Deng

Table: 1		
Antibody for immunohistochemistry	Source/Catalog number/Host/Isotype	Dilution
Nestin	R&D, MAB1259 Mouse, IgG	1:500
Nestin (human)	STEMCELL Technologies clone 10C2, mouse monoclonal IgG1 against human, cynomolgus nestin	1:200
Sox2	Gene Tex N1C3	1.200
NG2	Millipore AB5320 Rabbit IgG	1:20
Pax6	GeneTex, GTX113241 Rabbit IgG	1:500
Oct4	Gene Tex GT486 Rabbit IgG	1:200
S100 Beta	Sigma S2532 Mouse IgG	1:1000
GFAP	Millipore AB5804 Rabbit IgG 1:1000	1:1000
A2B5	Millipore MAb312 Mouse IgM 1:200	1:200
PDGFR alfa	R&D, MAB322, Mouses IgG	1:50
NG2	Millipore, AB5320, Rabbit IgG	1:200
Sox10	Gene Tex GSTX57197 Mouse IgG2b	1:300
Olig1	Phosphosolutions/1537 Rabbit IgG	1:200
Olig2	Phosphosolutions 1538, Rabbit IgG	1:1000
O4	MilliPore Sigma MAB345 IgG	1:200
O1	MilliPore Sigma MAB344 IgG	1:500
MBP	Millipore MAB386 Rat IgG	1:100
SMI312	Biolegend Mouse IgG1	1:200
GALC	Santa cruz, mouse (2D1) SC-293200 IgG_{2A}	1:200

Table: 2 RT-PCR probes used to characterize human NSCs.			
RT-PCR primer	Amplicon size	Taqman primer assay ID	Source
Nestin (Human)	81 bp	Hs00707120_s1	ThermoFisher Scientific
Sox2 (human)	91	Hs01053049_s1	ThermoFisher Scientific
Pax6 (human)	86	Hs01088114_m1	ThermoFisher Scientific
Sox1 (human)	96	Hs01057642_s1	ThermoFisher Scientific
GAPDH (human)	157	Hs02786624_g1	ThermoFisher Scientific

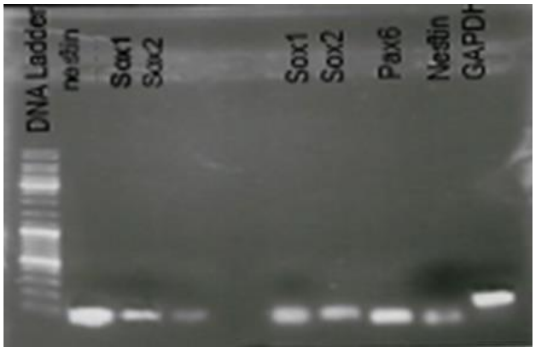

Figure A: RT-PCR gel data of BG01 and iPSC derived NSCs at passage 5. Lane 1-DNA ladder, lane 2-4 BGO1 derived NSCs, lane 6-10 iPSC derived NSCs. lane 5 is empty.

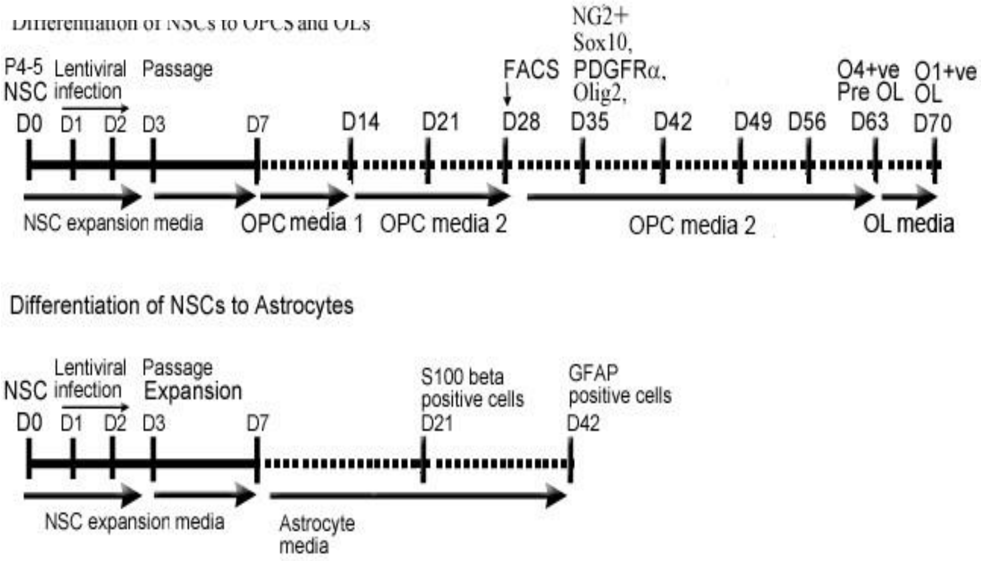

Figure B: Timeline for the development of zfp488 NSCs and their differentiation.

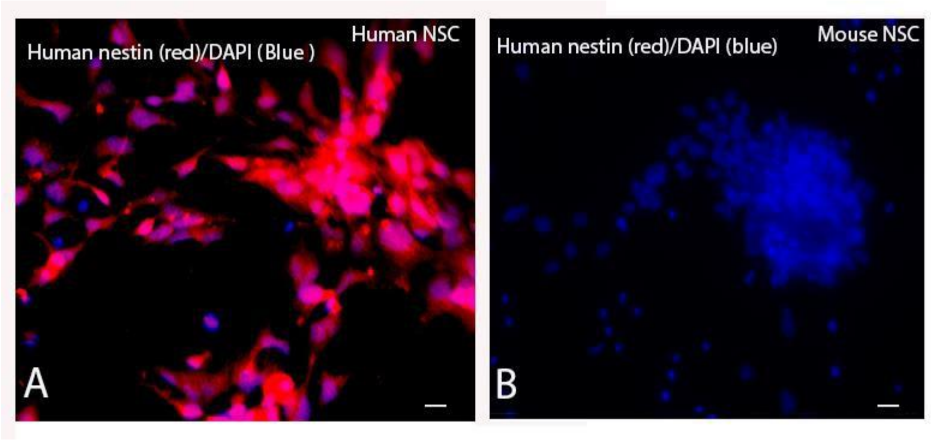

Figure C: (A) Anti human nestin antibody showed rubust staining (red) in human hESC derived NSCs. (B) Anti human nestin antibody did not cross react with mouse hESC derived NSC.

METHODS

Generation of LPC induced demyelination in RAG-2 KO mouse corpus callosum: Young adult mice (8-10 weeks old) were anesthetized with 5% isoflurane in 100% oxygen with a delivery rate of 5 L/min. Later, anesthesia was maintained with 1.4–2.0% isoflurane in 100% oxygen with a flow of 1 L/min. The head of the mice were secured in a stereotaxic instrument (David Kopf, Tujunga, USA). The skin was disinfected with 0.1% Chlorhexidine gluconate solution, followed by 70 % ethyl alcohol, and cut with a sharp scalpel. A burr hole was drilled at the following stereotaxic coordinates: anteroposterior 0.2; mediolateral 1.1 with Bregma as reference; and dorsoventral 2 mm from skull surface. For induction of demyelination, 1 µl of 1% LPC (Sigma-Aldrich, CA, USA) was injected aseptically through the burr hole into the corpus callosum using a Nanoject II microinjector (Drummond Scientific company, USA). Postoperative care was given according to approved IACUC protocols of University of California, Davis.

hNSC injections into corpus callosum: 48 hours after the LPC injections, mice were anesthetized again, and a burr hole was drilled 0.4 mm lateral from LPC injection site. 100,000 zfp488-hNSCs or control-NSCs were injected very carefully in a volume of 2 microliter (in sterile PBS) using a Nanoject II microinjector (Drummond Scientific company, USA). Injection duration was 3 min, after which the needle was left in place for 3 minutes before withdrawing very slowly.

Myelination medium (Rat DRG -OPC coculture): 39 ml of DMEM-High Glucose medium + 400 µl each of insulin stock, 100X L-glutamine (Invitrogen)+ 100X sodium pyruvate (Invitrogen) + Add 200 µl each of 200X Hormone mix (see below), and 40 µg/ml T3 + 40 µl each of 50 µM hydrocortisone (Sigma) +8 µl each of 5000X biotin stock and 5000X Vitamin B12 + 15 µl B27 Supplement (Invitrogen). The media was filtered through a 0.22-µm filter.

Hormone cocktail (200X): Dissolved 20 mg apotransferrin (Sigma) in 5 ml DMEM/F12. Dissolved 64 mg putrescine (Sigma) in 5 ml DMEM/F12. Mixed these two solutions. To this added 10 µl progesterone (Sigma; from stock: 2.5 mg /100 µl ethyl alcohol)+ 51.9 µl sodium selenite (Sigma; from stock: 4.0 mg+10 µl 1N NaOH in 10 ml DMEM) + and 10 ml DMEM for a final stock concentrations of 1 mg/ml

apotransferrin, 20 mM putrescine, 4 µM progesterone, and 6 µM selenium . Filtered through 0.22 µm filter. Made 100 µl aliquots; stored at -20˚C.

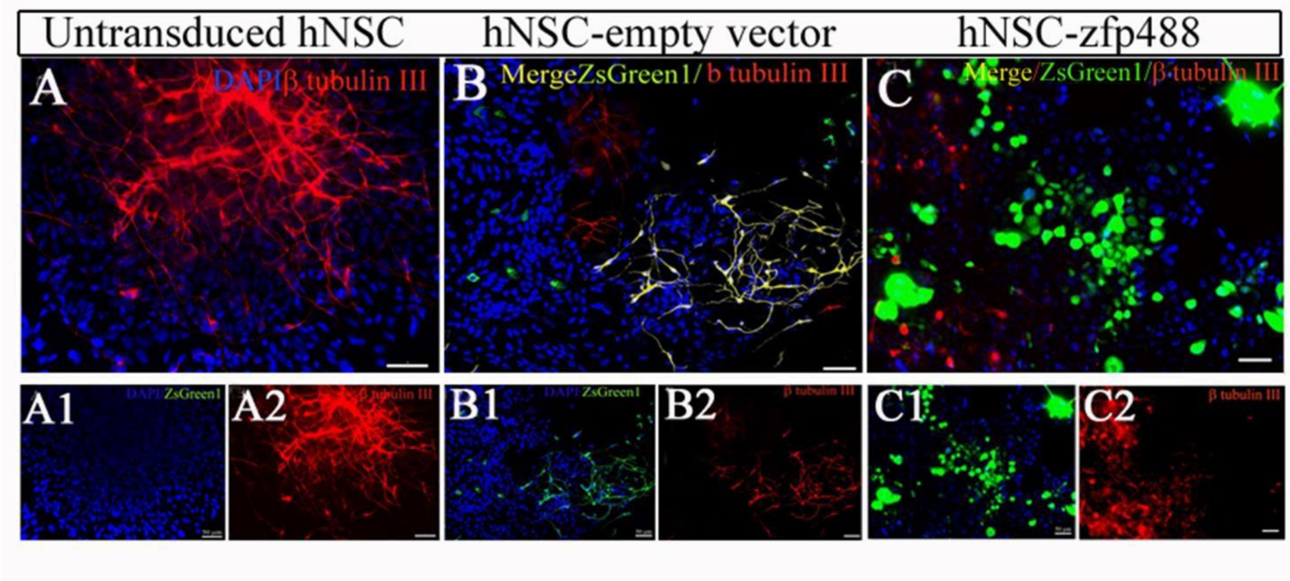

Supplementary figure 1: Expression of zfp488 during spontaneous differentiation of H9 hESC derived hNSCs prevented their default neuronal fate in vitro: Following 3 week of spontaneous differentiation, majority of the untransduced (suppl Fig. 1A) and control hNSCs (ZsGreen positive cells, Fig. 2B) differentiated into beta III tubulin positive immature neurons. In contrast, zfp488+/ZsGreen positive green cells rarely differentiated into beta tubulin positive neurons (suppl Fig. 1C). Thus, expression of zfp488 prevented the hNSC from acquiring their default fate of differentiating into neurons after removal of growth factors.

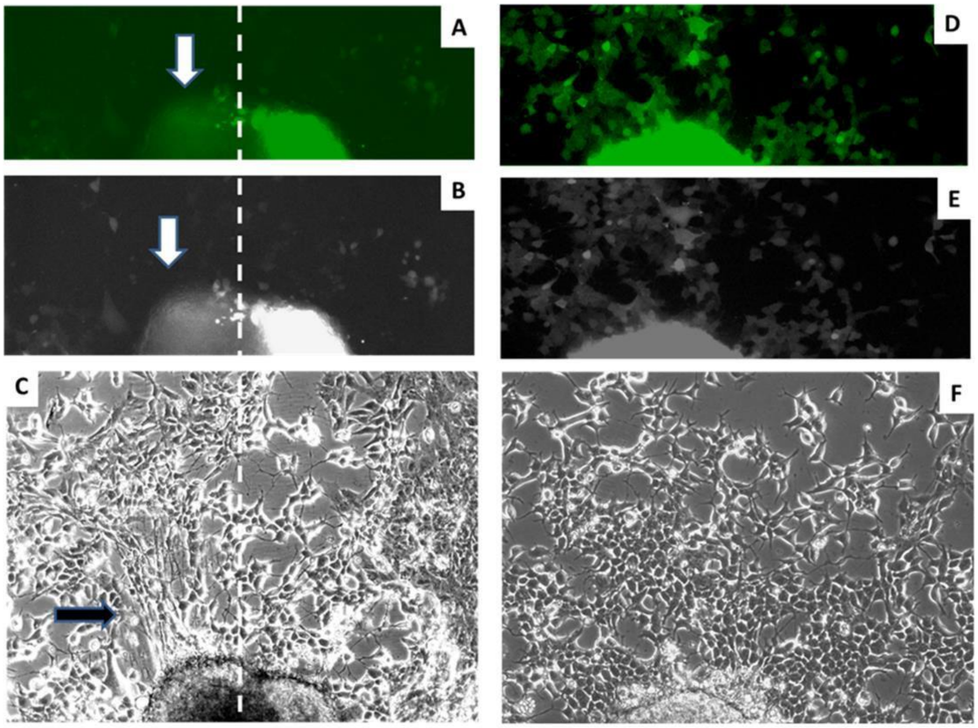

Supplementary figure 2: Suppression of neuronal differentiation in zfp488 expressing NSCs derived from BGO1 HESCs. (A) A BG01 derived zfp488-NSC neurosphere was selected from sphere culture, with its left side not expressing Zsgreen/ zfp488 (not green); while the right side was expressing ZsGreen /zfp488 (green) (B is the phase picture of the same sphere). (C) 48 hours after plating the neurosphere, neurons appeared from the non-green side (arrow) of the sphere, while glial progenitor like small cells mixed with undifferentiated NSC like cells started to come out of the ZsGreen side of the spheres. (D-F) Similarly, in an iPSC derived zfp488-NSC neurospheres, 48 hours after plating in a neuronal differentiation media, most cells were undifferentiated NSC or glial progenitor morphology, no neurons were seen emerging.

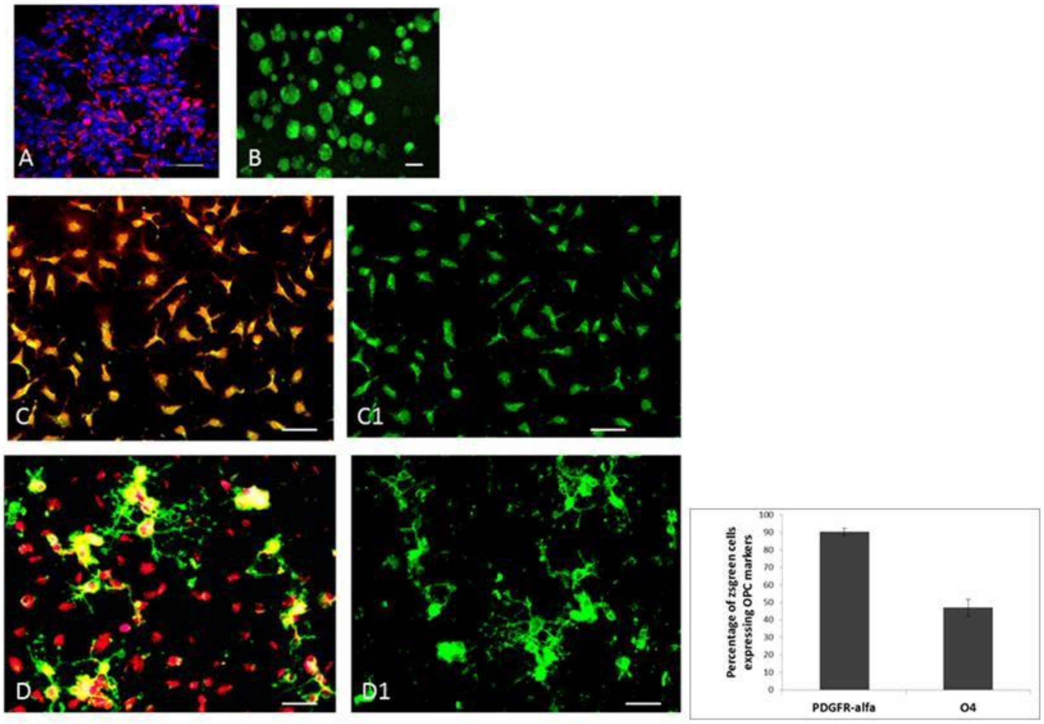

Supplementary Figure 3: Oligodendrocyte differentiation of BGO1 hESC derived zfp488-NSCs. (A) Nestin positive zfp488-NSCs were generated by the method described earlier. (B) zfp488 positive NSC were cultured as spheres in OPC media for two weeks, then the Zsgreen positive spheres were as a plated as monolayer and cultured for 1 more week. (C) At 3 weeks, 90.3 $\pm$ 1.9% Zsgreen cells (C1) were PDGFR alfa positive (red, overlay is yellow), and at 4 weeks (D-D1), 47.0 $\pm$ 4.7% Zsgreen cells were O4 positive (red). Overlay is yellow. Scale bar-50 micron.

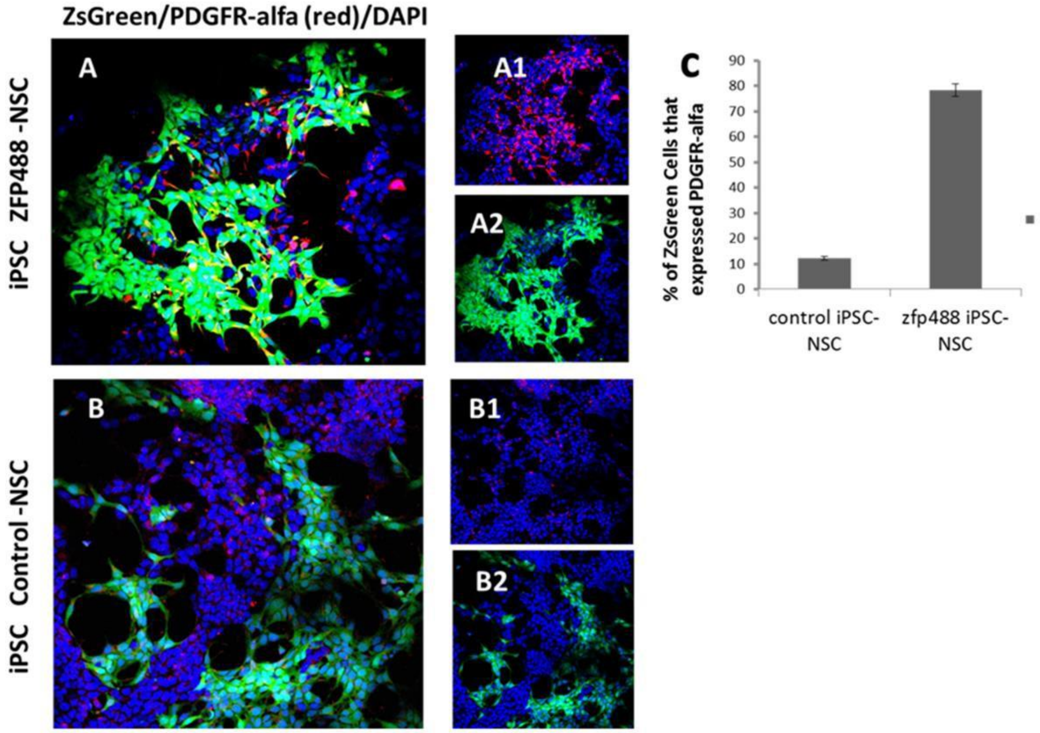

Supplementary figure 4: Robust generation of PDGFR-alfa positive OPCs from iPSC derived zfp488-NSCs. (A) After culturing in O.PC induction media, majority of iPSC derived Zsgreen/zfp488 positive NSC (green) differentiated into PDGFR-alfa expressing (red) OPCS, while less than ten percent of control NSCs expressed PDGFR-alfa. Because of low density OPCs did not survive later. (C) Quantification of immunohistochemistry data. Scale bar-50 micron. Scale bar-50 micron

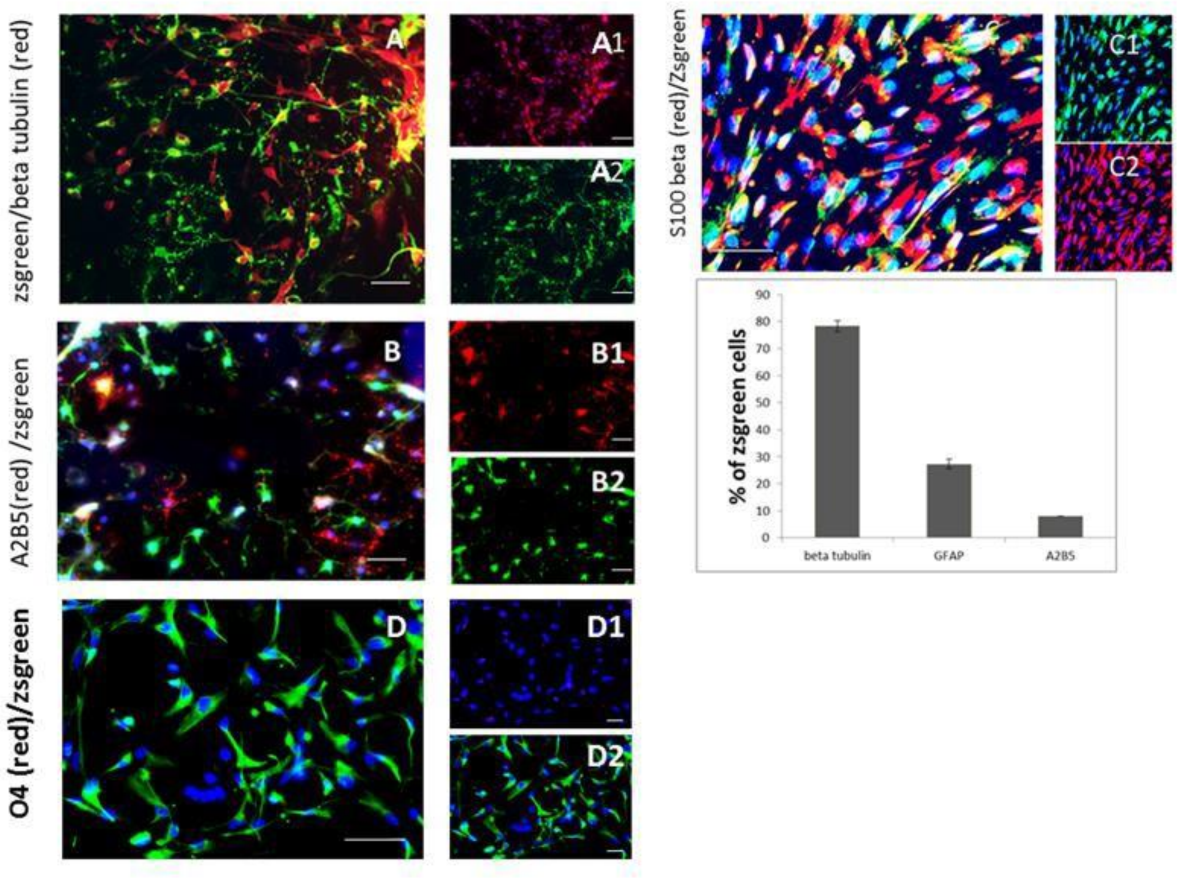

Supplementary figure 5: Predominant neuronal fate of control iPSc derived NSCs. iPSC derived control Zsgreen-NSCs primarily differentiated into neurons and astrocytes. After withdrawing growth factors and culturing in neuronal media, majority of control NSC s (78.3 + 2.0%) differentiated into beta tubulin III positive neurons (A-A2). Control NSCs cultured in OPC media1 and OPC media 2, had nominal differentiation into A2B5+ cells (red, 8.0 + 1.6%). Majority of the Zsgreen (C2) cells differentiated (27.3 + 1.8%) into S100 beta positive cells (red, C1). (D) No O4 positive cells were detected. (E) Quantitation of the percentage of Zsgreen cells that coexpressed neuronal, OPC, or astrocyte markers. Scale bar-50 micron.

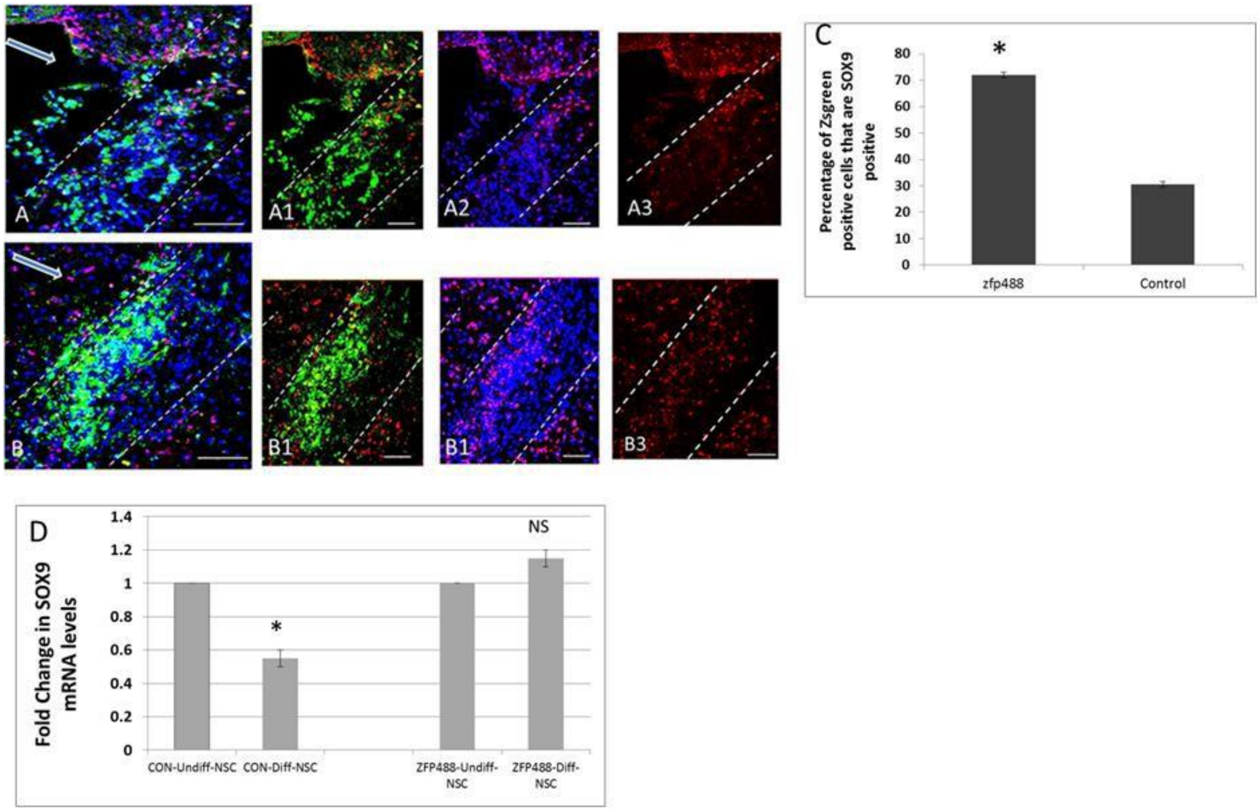

Supplementary figure 6: (1) significantly greater percentages of sox9 co-expressing cell were seen in the Zsgreen/zfp488 NSCs group compared to the Zsgreen/control NSCs in the mouse corpus callosum one week after transplantation. (A-A3) Co-expression of sox9 (red) in Zsgreen cells 1week after transplantation of control-NSCs in the corpus callosum of mice (arrow shows needle track). (B-B3) Co-expression of Zsgreen and sox9 (red) after transplantation of zfp488-NSCs in the corpus callous (arrow shows needle track). C) The graph shows the quantification of immunohistochemistry data, (N=3). The percentage of Zsgreen cells co-expressing Sox9 was double in zfp488-NSC group compared to control NSCs (P<0.001). (2) The Sox9 mRNA levels in the zfp488-NSCs were remained unchanged following differentiation. D)Quantification of mRNA expression levels of Sox9 after 1 week of differentiation of NSCs. Sox9 mRNA levels were significantly reduced (P<0.001) in the cells derived from differentiation of control NSCs (these cells were primarily neurons), while Sox9 mRNA levels were maintained in differentiating cells derived from zfp488 NSCs. N=3 for each group. Scale bar-50 micron.
